# Ionizable guanidine-based lipid nanoparticle for targeted mRNA delivery and cancer immunotherapy

**DOI:** 10.1126/sciadv.adx5970

**Published:** 2025-10-24

**Authors:** He Zhang, Dejing Liu, Kang Yang, Zhuoying Liang, Mao Li

**Affiliations:** Institute of Chemical Biology, Shenzhen Bay Laboratory, Shenzhen, China.

## Abstract

The development of lipid nanoparticle (LNP) systems has largely advanced RNA therapeutics, particularly mRNA-based cancer immunotherapy. Conventional amine-LNPs, designed for hepatic RNA delivery, face challenges in targeting lymphoid organs effectively and maximize antigen presentation. In this study, we present the development of pH-responsive ionizable guanidine-LNPs (G-LNPs). Our cholesterol-free G-LNP system enables efficient delivery of mRNA to the spleen following intravenous administration. Notably, while both amine-LNPs and G-LNPs can deliver mRNA to the spleen, G-LNPs exhibit a unique ability to preferentially target antigen-presenting cells, leading to significantly enhanced antigen presentation and robust T cell activation. mRNA vaccines formulated with G-LNPs elicited strong and antigen-specific immune responses, providing complete protection against tumor progression. In addition, intraperitoneal administration of G-LNPs enabled selective mRNA expression in the pancreas, showcasing the versatility of this delivery platform. These findings underscore the potential of guanidine-LNPs as a highly promising platform for organ-targeted mRNA delivery and cancer immunotherapy.

## Teaser

Ionizable guanidine-based LNP achieves extrahepatic mRNA delivery and exhibits enhanced ability to activate immune cells.

## INTRODUCTION

The advancement of lipid nanoparticle (LNP) delivery technology has been a pivotal step forward in the field of RNA therapeutics (*1*–*7*). This is particularly evident in the successful use of mRNA vaccines against the severe acute respiratory syndrome coronavirus 2 pandemic, highlighting the effectiveness of LNP over other delivery methods. This success has led to the development of versatile LNP systems targeting a wide range of diseases including infectious diseases, genetic disorders, and cancers (*8*–*10*). Among these, mRNA-based cancer immunotherapy has emerged as a particularly promising treatment strategy (*11*–*18*). By using LNP platforms to deliver mRNA encoding for tumor-specific antigens, these therapies can induce robust immune responses that specifically target and eliminate malignant cells.

An essential prerequisite for effective cancer immunotherapy is the ability to deliver mRNA vaccines specifically to immune-related organs such as the spleen, where potent antitumor immune responses are initiated (*19*–*21*). Key to this process is the efficient translation of the antigen-encoding mRNA and its subsequent presentation to immune cells, which are critical for achieving therapeutic efficacy (*22*–*27*). Conventional LNP formulations, however, predominantly employ amine-based lipids (e.g., secondary and tertiary amines), which are optimized for efficient intracellular mRNA release through pH-responsive protonation in acidic endosomal environments (*8*, *28*–*31*). While this design effectively facilitates hepatic mRNA delivery (*32*), it limits the systemic delivery of mRNA to immune-related organs and cells (*9*, *33*–*36*). Consequently, spleen-targeted delivery and optimal antigen presentation by immune cells often remain suboptimal in conventional amine-based LNP systems. Moreover, many attempts to redirect amine-based LNPs to immune-related organs have largely focused on modifications for organ-level targeting (*37*–*50*), with less emphasis on improving cellular translation and antigen presentation.

These challenges highlight an urgent need for innovative lipid systems that enable efficient mRNA delivery to immune organs while enhancing immune activation. In this regard, guanidine-based lipids present a compelling yet underexplored alternative to amine-based systems. While both guanidinium and ammonium functional groups use electrostatic interactions to bind anionic biomolecules, guanidinium offers an additional advantage by forming bidentate hydrogen bonds (*51*–*53*). This unique chemical feature improves binding affinity with negatively charged species, such as mRNA and cell membranes, and has been shown to enhance cellular uptake and delivery efficiency in other biomaterials, including polymers (*54*–*56*), peptides (*57*–*59*), and nanoparticles (*60*, *61*). Notably, guanidine-functionalized nanoparticles have demonstrated superior antigen uptake and enhanced dendritic cell (DC) activation, which are pivotal for immune responses (*62*, *63*). However, the potential of guanidine-based carriers for in vivo mRNA delivery has been largely unrealized due to challenges associated with their permanently positive charge [guanidinium p*K*_a_ (where *K*_a_ is the acid dissociation constant) ~ 13], which can result in nonspecific interactions with other biomolecules and increase systemic cytotoxicity.

**Fig. 1.**
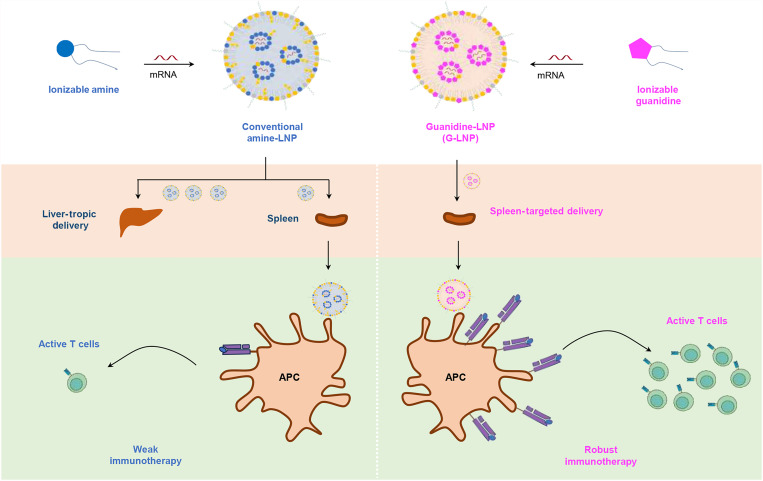
Schematic representation of the conventional amine-based LNP and guanidine-based LNP (G-LNP). G-LNP can elicit antigen-specific immune responses, resulting in substantially enhanced T cell activation and cancer immunotherapy.

To overcome these limitations, we propose the rational design of ionizable guanidine-based lipids that retain the advantageous anion-binding properties of guanidinium while adopting a neutral or negative charge at physiological pH. This pH-responsive behavior enables effective mRNA delivery while minimizing systemic toxicity. Leveraging these properties, we have developed guanidine-based LNPs (G-LNPs) as a platform for efficient in vivo mRNA delivery (Fig. 1). Among these, a cholesterol-free, three-component G-LNP system demonstrated remarkable specificity for spleen targeting following intravenous administration. G-LNPs achieved functional delivery of antigen-encoding mRNA to antigen-presenting cells (APCs), driving potent immune responses that provided exceptional tumor protection. Furthermore, G-LNPs facilitated efficient mRNA expression in other tissues, such as the pancreas, when administered via intraperitoneal injection, underscoring their versatility and organ-specific delivery potential. Therefore, guanidine-based LNPs represent a promising platform for organ-targeted mRNA delivery and cancer immunotherapy.

## RESULTS

**Fig. 2.**
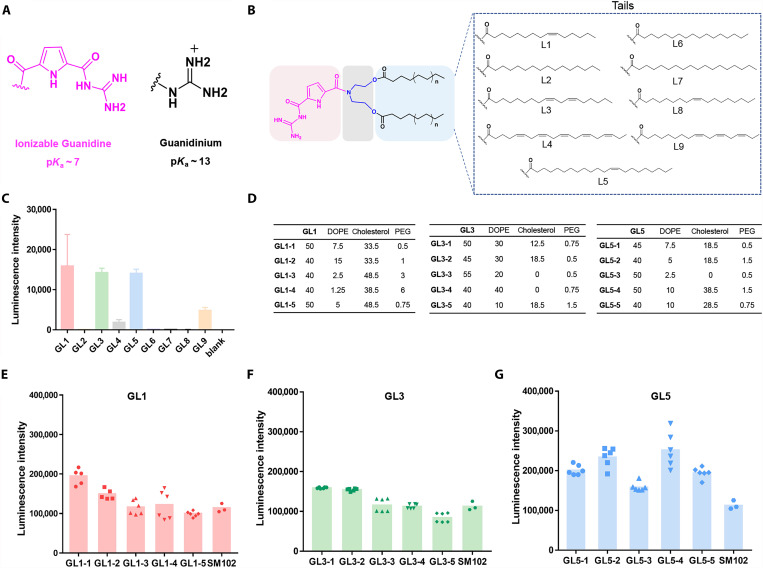
G-LNPs exhibit enhanced mRNA delivery performances in vitro. (**A**) Chemical structure and properties of the GCP unit and guanidine unit. (**B**) Chemical structures of the GL-lipids used in the study. (**C**) mRNA delivery efficiency of different GL-lipids. (**D**) Five most efficient LNP formulations for GL1, GL3, and GL5. (**E** to **G**) Luciferase mRNA expression levels generated from the in vitro mRNA delivery experiments of GL1, GL3, and GL5 in HeLa cells (150 ng mRNA per well). SM102 was applied as a positive control.

We began our investigation with the design and synthesis of guanidino-carbonyl-pyrrole (GCP) functionalized lipids (GL lipids). The GCP unit can form multiple hydrogen bonds with oxoanions (Fig. 2A) and has been applied to modify peptide and polymers to achieve efficient DNA transfection in vitro (*64*). Moreover, the low p*K*_a_ value of the GCP unit (~7) enables its pH-responsive behavior under acidic conditions, such as those found within cells (*65*, *66*). A total of nine lipids equipped with a GCP headgroup were prepared through a two-step synthetic route. The hydrophobic tails were attached to the headgroups via an ester linkage (Fig. 2B). We initially screened for the most effective lipids using a traditional four-components LNP formulation with firefly luciferase mRNA in HeLa cells. The results indicated that lipids GL1/GL3/GL5 demonstrated superior mRNA delivery ability compared to other lipids (Fig. 2C). Therefore, we further optimized the molar ratio between each component using an orthogonal screening approach developed by Li *et al.* (*67*). By systematically altering the components in the formulation, we screened 32 formulations for GL1, GL3, and GL5 (fig. S1 and tables S1 to S3) and found several formulations that exhibited better mRNA delivery efficiency than SM102, the current benchmark LNP in mRNA delivery. Among these, lipids with longer tails, i.e., GL5 LNPs, showed the best performances, with several formulations exhibiting more than twofold higher efficiency than SM102 LNP (Fig. 2, D to G). Notably, the excellent mRNA delivery ability of these LNPs could also be observed in other cell lines such as U251 and MB231 cells (fig. S2). Unexpectedly, we found that even in the absence of cholesterol, i.e., in three-component LNPs, formulations GL3-3, GL3-4, and GL5-3 demonstrated remarkable mRNA delivery abilities, with efficiency comparable to or exceeding that of SM102. In addition, we also studied the impact of the weight ratio between LNP and mRNA on delivery efficiency. The results demonstrated that the best performance was achieved with a 40:1 weight ratio (fig. S3). This lipid/RNA ratio was thus used in the following experiments.

**Fig. 3.**
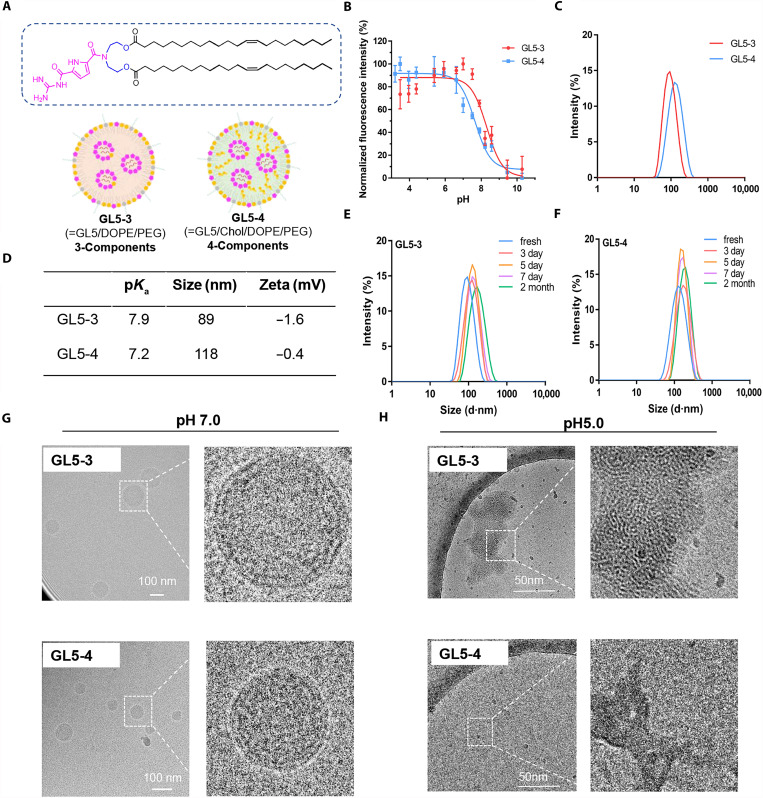
GL5 LNPs demonstrate optimal physiochemical property and pH responsiveness. (**A**) Schematic representation of the three-components GL5-3 and four-component GL5-4 LNPs. (**B**) TNS fluorescence assays and (**C**) DLS size distribution profiles for GL5-3 and GL5-4. (**D**) Table of the formulations for the selected LNPs and their corresponding p*K*_a_, size, and zeta potential profiles. (**E** and **F**) Size distribution of GL5 LNPs after storage at 4°C. (**G** and **H**) Cryo-EM images of GL5-3 and GL5-4 LNPs at different pH levels.

We then measured the apparent p*K*_a_ and size for the selected LNPs using 2-(p-toluidino)-6-napthalene sulfonic acid (TNS) fluorescence assays and dynamic laser scattering (DLS). Both GL1 and GL3 LNPs showed a similar p*K*_a_ profile, ranging from 6.1 to 6.7 (fig. S4). The presence of cholesterol did not notably affect the size distribution of both LNPs. For example, GL3-3 and GL3-4 lipids formed LNPs of ~200 nm in average size without cholesterol. When cholesterol was added to the formulation, the size slightly increased to about 230 nm (fig. S4, GL3-1). In contrast, GL5 LNPs showed higher p*K*_a_ values, ranging from 7.0 to 7.9 (Fig. 3B). At the same time, GL5 LNP had a much smaller size compared to other lipids. Depending on the presence of cholesterol, the average size of GL5 LNPs in solution ranged from 89 to 118 nm (Fig. 3C), which is more than half the size of the other LNPs. The differences in size between GL5 and other LNPs suggest a more compact packing mode for GL5 lipid. In addition, these LNPs showed a slightly negatively charged surface in pH 7.4 phosphate-buffered saline (PBS) buffer, further confirming the neutral p*K*_a_ of the GCP head group. The cholesterol-free three-component GL5-3 LNP had a comparable stability to that of the four-component GL5-4 LNP at 4°C within 1 week. However, a slightly increase in the size was observed for GL5-3 LNP after storage for 2 months (Fig. 3, E and F).

Examining the morphology of GL5 LNPs under physiological condition using cryo–electron microscopy (cryo-EM) revealed that these G-LNPs displayed a multilayered structure in solution (Fig. 3G). The presence of cholesterol, however, did not change the overall structure of these LNPs. Prior research has indicated that GCP functionalization can facilitate molecular self-assembly to form nanoparticles and nanofibers via the stacking interaction between GCP units (*64*). Consequently, it is plausible that GL-lipid behaves in a similar manner, combining with the hydrophobic interactions in the tail regions, thus stabilizing the formation of LNPs without the necessity for cholesterol. Under acidic conditions, the nanoparticle structures of G-LNPs were completely disassembled and rearranged to irregularly aggregates, as observed by cryo-EM in pH 5.0 buffer (Fig. 3H). This high pH sensitivity of G-LNPs could be beneficial for the intracellular release of mRNA once they enter into the acidic environments of endolysosomes.

**Fig. 4.**
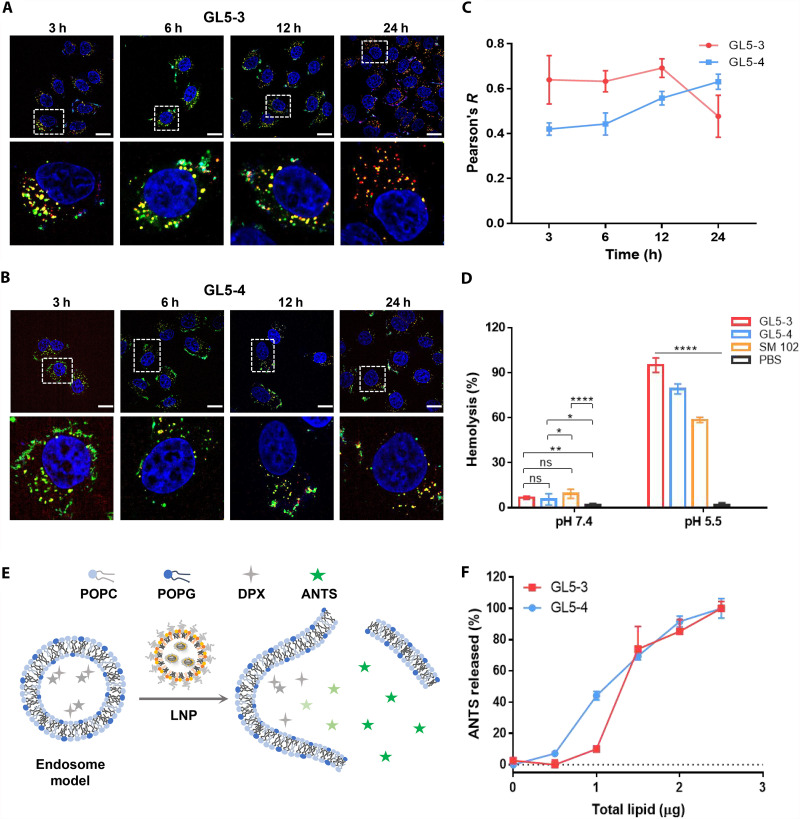
Endosomal escape properties of GL5 LNPs. (**A** and **B**) Merged confocal microscopy images of the cells treated with Cy5-labeled mRNA (red) together with GL5-3 (A) and GL5-4 (B) at indicated time points, respectively. Cell nuclei were stained with Hoechst (blue), and lysosomes were stained with lysotracker (green). Scale bars, 20 μm. (**C**) Pearson’s colocalization coefficients of Cy5-mRNA and lysosomes at indicated time points. (**D**) Hemolysis assays of LNPs in pH 7.4 or 5.5 buffers. (**E**) Schematic representation of the ANTS release assay. POPC/POPG LUVs are used as endosomal membrane model. (**F**) ANTS release assays for GL5-3 and GL5-4 LNPs with POPC/POPG LUVs in pH 5.0 citrate buffer. The amount of total lipid represents the sum of each component in respective LNPs. ns, *P* > 0.05.

To better understand the cellular trafficking of GL5 LNPs, we tracked the cellular uptake process using mRNA labeled with Cy5 in living cells. Confocal microscopic examinations revealed that a large amount of RNA was delivered into the endolysosomal compartments of cells within the first 12 hours by both types of LNPs, irrespective of the content of cholesterol. However, a substantial portion of mRNA was released into the cytosol for the cholesterol-free LNP GL5-3 after 24 hours (Fig. 4, A to C).

We therefore investigated whether these G-LNPs could destabilize endosomal membranes in an acidic buffer using an ANTS (8-aminonaphthalene-1,3,6-trisulfonic acid) release assay (*68*). We used a large unilamellar vesicle (LUV) constructed from a combination of lipids 1-palmitoyl-2-oleoyl-*sn*-glycero-3-PC (POPC)/1-palmitoyl-2-oleoyl-*sn*-glycero-3-phosphatidylglycerol (POPG) as a model for the endosomal membrane (*69*). ANTS and its fluorescent quencher DPX (*p*-xylene-bispyridinium bromide) dyes were encapsulated inside the LUVs. In a pH 5.0 buffer, the addition of GL5 LNPs into the LUV solutions resulted in the release of ANTS and subsequently an increase in the fluorescence signal (Fig. 4, E and F). Both types of G-LNPs could effectively induce ANTS release. LNPs formulated with cholesterol demonstrated slightly stronger ability to release the encapsulated ANTS dye at lower concentrations. Nonetheless, the difference was negligible when the amount of lipids exceeded 1.5 μg, suggesting an equally effective destabilization of LUV membranes by these lipids. This mechanism could be used by these G-LNPs inside the acidic endolysosomes, thus facilitating the release of mRNA into the cytosol. Moreover, we assessed the membrane-disrupting capability of G-LNPs at different pH levels using a hemolysis assay. Both GL5-3 and GL5-4 demonstrated minimal membrane-disrupting activity under physiological pH, similar to SM102. However, the three-component GL5-3 exhibited more than 90% membrane-lysis efficiency at acidic pH (Fig. 4D). The incorporation of cholesterol into the formulation slightly reduced the membrane-lysis ability of the LNPs. Nevertheless, the enhanced membrane-disrupting activity of GL5 under acidic conditions underscores the significance of the protonation state of ionizable lipids. The guanidine groups in G-LNPs are electrically neutral at pH 7.4 (*64*). Upon enter into acidic endosomes (pH 4.5 to 6.5), these groups undergo protonation and acquire positive charges. These protonated guanidine lipids can interact more efficiently with anionic species on the endosomal membrane compared to amine-based lipids, thereby facilitating the release of mRNA into cytosol (*70*).

**Fig. 5.**
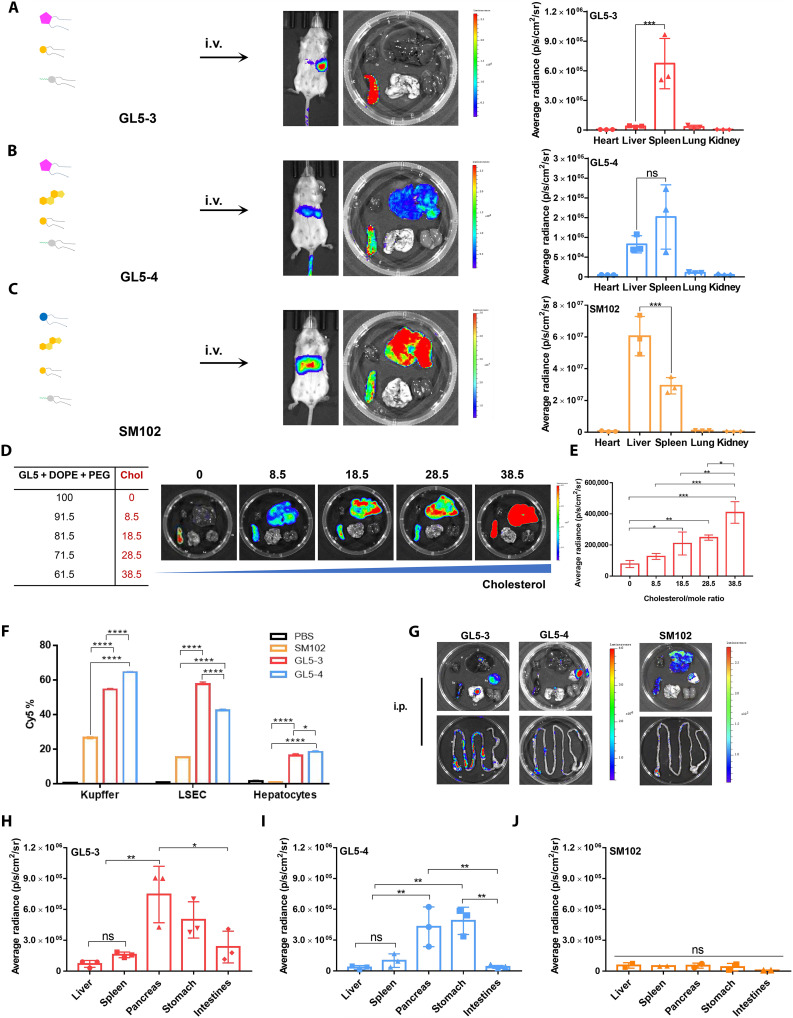
GL5 LNPs achieve targeted mRNA delivery in vivo. (**A** to **C**) Schematic representations of the three-component GL5-3, four-component GL5-4, and SM102-LNPs and evaluation of their mRNA delivery properties. Balb/c mice were intravenous (i.v.) injected with LNPs at the Luc mRNA dose of 0.2 mg/kg. (**D** and **E**) Biodistribution and quantification of GL5 LNPs with varying amount of cholesterol. (**F**) Percentage of Cy5^+^ liver cells for the mice treated with G-LNP or SM102 LNP. (**G**) Biodistribution and quantitative analysis (**H** to **J**) of intraperitoneally (i.p.) injected LNPs loaded with luc mRNA (0.2 mg/kg). H, heart; Li, Liver; Sp, Spleen; Lu, Lung; Ki, Kidney; Pa, Pancreas; In, Intestine; St, Stomach. Statistical analysis was performed with analysis of variance (ANOVA) test (*P < 0.05, **P < 0.01, ***P < 0.001, and ****P < 0.0001), *n* = 3.

We proceeded to assess the effectiveness of these LNPs in vivo by injecting them intravenously into mice. Among these, GL1-LNP and GL3-LNP could only deliver functional luciferase mRNA to the injection site (fig. S5). In contrast, GL5-LNPs successfully delivered functional luciferase mRNA to distal organs, highlighting their systemic delivery potential. Both the cholesterol-free GL5-3 and cholesterol-containing GL5-4 LNPs led to mRNA expression in mice, as visualized through luciferase activity (Fig. 5, A and B). Among them, GL5-3 demonstrated excellent spleen-targeting ability, as confirmed by quantification of radiance intensity and luciferase activity directly in isolated organs (fig. S7). Furthermore, this spleen-targeting property remained largely intact across varying ratios of GL5 lipid to the helper lipid DOPE (1,2-dioleoyl-*sn*-glycero-3-phosphoethanolamine), reinforcing the targeting ability of GL5-3 (fig. S8). By contrast, SM102, an amine-based LNP widely used for mRNA delivery, predominantly directed the mRNA cargo to the liver and less in the spleen (Fig. 5C). Notably, GL5-3 displayed a slightly negative surface potential (−1.6 mV). As previously reported, negatively charged LNPs are favored for spleen-targeted mRNA delivery (*71*). Furthermore, the lack of cholesterol in GL5-3 may further facilitate extrahepatic mRNA delivery. Cholesterol is known to promote the adsorption of apolipoprotein E (ApoE) onto the LNP surface. Increased cholesterol content could enhance ApoE adsorption, which in turn mediates internalization through low-density lipoprotein receptors on hepatocyte membranes (*72*).

Whereas the cholesterol-free GL5-3 selectively targeted the spleen, introducing cholesterol into the formulation (GL5-4) altered its biodistribution. GL5-4 delivered mRNA to both the spleen and liver at comparable levels, largely negating its spleen specificity (Fig. 5B). The extent of liver-associated mRNA expression strongly correlated with the cholesterol content, as formulations with higher cholesterol concentrations showed increased mRNA accumulation in the liver (Fig. 5, D and E). This observation aligns with findings by Su *et al.* (*73*), which demonstrated that cholesterol removal could shift mRNA delivery targets for amine-based LNPs from the liver to other organs. Our study further underscores that cholesterol-free formulations are advantageous for targeting extrahepatic sites, such as the spleen.

Next, we explored the in vivo trafficking and organ distribution properties of these guanidine-based LNPs using 1,1′-Dioctadecyl-3,3,3′,3′-Tetramethylindotricarbocyanine Iodide (DiR)-labeled LNPs (*74*). Mice were injected with DiR-labeled LNPs encapsulating luciferase mRNA and individual organs were harvested after 6 hours. Cholesterol-containing GL5-4 accumulated in both the liver and spleen, a pattern comparable to that of SM102 (fig. S8). In contrast, the removal of cholesterol reduced liver accumulation for GL5-3, indicating that cholesterol depletion can indeed influence targeting performance. Further analysis of liver cell distribution using Cy5-labeled mRNA revealed that G-LNPs preferentially delivered mRNA to liver sinusoidal endothelial cells (LSECs) and Kupffer cells—key players in hepatic immune regulation—at notably higher levels than SM102 (Fig. 5F) (*75*, *76*). Together, these findings suggest that cholesterol-free formulations such as GL5-3 mitigate liver accumulation, thereby enhancing spleen delivery, while cholesterol-containing formulations such as GL5-4 drive dual targeting characteristics.

We next explored how administration routes influence the delivery efficiency and targeting properties of G-LNPs. Strikingly, both GL5-3 and GL5-4 delivered luciferase mRNA to the pancreas and stomach via intraperitoneal injection (Fig. 5, G to J). GL5-3 also efficiently transported mRNA to the intestine, while GL5-4 was ineffective. By contrast, SM102 exhibited minimal mRNA delivery after intraperitoneal administration, emphasizing the versatility of guanidine-based LNPs for multiple administration routes.

**Fig. 6.**
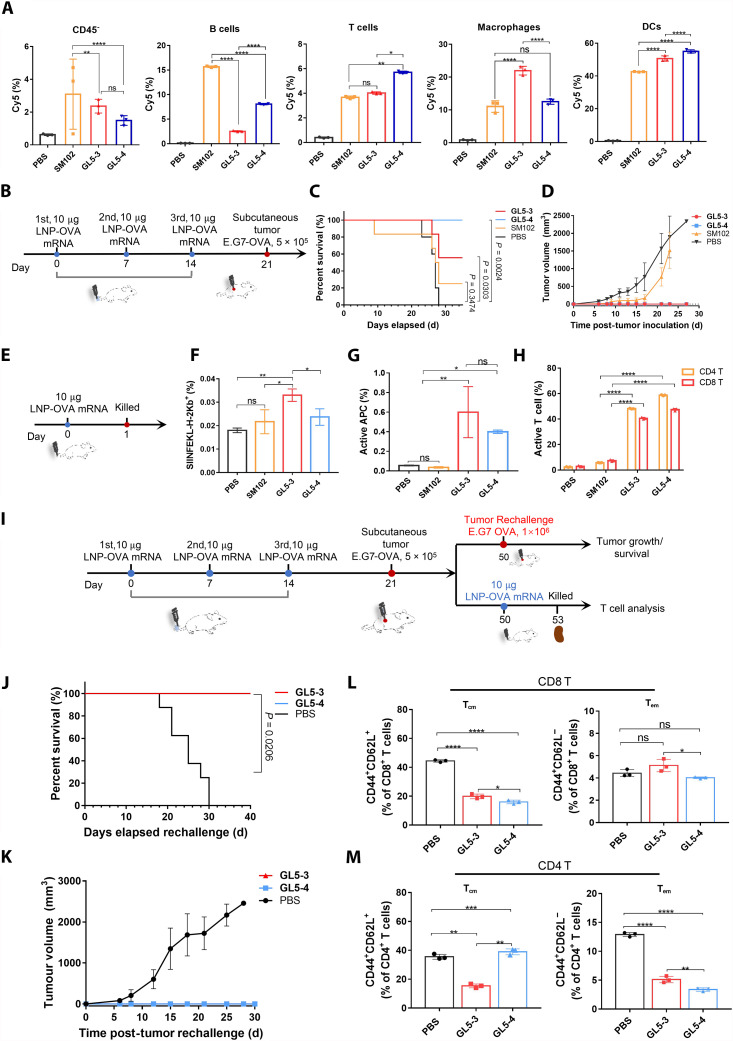
GL5 LNPs induce robust antitumor immune responses. (**A**) Percentage of Cy5^+^ splenic cells for the mice intravenously injected with G-LNP or SM102-LNP. Mice: *n* = 3 for each group. (**B**) Schematic representation of the vaccination model in C57BL/6 mice. Mice were vaccinated with three doses of OVA-mRNA-LNPs at 1-week intervals before subcutaneous inoculation of OVA-expressing T lymphoma (E.G7-OVA) cells. Mice: *n* = 5 for each group. (**C**) Survival curves and tumor volumes (**D**) of vaccinated mice after tumor inoculation. (**E**) Schematic representation for the analysis of APC and T cells in the spleen after vaccination. Mice: *n* = 3 for each group. Splenocytes were isolated for analysis of CD45^+^CD11c^+^CD86^+^ (**F**) and SIINFEKL-H-2Kb^+^ (**G**) cells after 72 hours and CD69^+^ T cells after 24 hours (**H**). (**I**) Schematic representation of the rechallenge model. Thirty days after the first inoculation, 1 × 10^6^ E.G7-OVA cells were subcutaneously reinjected into the mice. Mice: *n* = 5 for each group. (**J**) Survival curves and tumor volumes (**K**) of the rechallenged mice. (**L** and **M**) Spleens of mice were analyzed by flow cytometry for CD8^+^ (L) and CD4^+^ (M) memory T cells. Statistical analysis was performed with ANOVA test (*P < 0.05, **P < 0.01, ***P < 0.001, and ****P < 0.0001).

Building upon the exceptional spleen-targeting capability of G-LNP, we further explored its potential for delivering cancer mRNA vaccines to the spleen to elicit antigen-specific immune responses. To assess its transfection efficiency in spleen-resident cell populations, we used Cy5-labeled mRNA as a reporter molecule. Notably, GL5-3 demonstrated significant efficacy in mRNA delivery to APCs, achieving transfection rates of ~51% in DCs and 22% in macrophages. GL5-4 showed even higher transfection rates in DCs, reaching 55%. These rates markedly exceeded those observed with SM102, which transfected 42% of DCs and 11% of macrophages, respectively (Fig. 6A). However, SM102 showed notably higher transfection rates in nonimmune cells and B cells. The enhanced delivery of mRNA and subsequent antigen expression by APCs, such as DCs, is vital for initiating robust and protective immune responses to tumor cells (*12*). These cells play an instrumental role in priming T cells by presenting antigen-derived peptides via major histocompatibility complex (MHC) molecules, further underscoring the promise of G-LNP as a platform for cancer immunotherapy.

These insights prompted us to encapsulate an ovalbumin (OVA)–encoding mRNA, a common model antigen, into various LNP formulations to evaluate their effectiveness as cancer vaccines. Experiments were conducted in a murine model, where C57BL/6 mice were vaccinated with LNPs formulated with GL5-3, GL5-4, or SM102 on days 0, 7, and 14. On day 21, the mice were challenged with E.G7-OVA tumor cells (5 × 10^5^ cells, subcutaneously; Fig. 6B). G-LNP formulations GL5-3 and GL5-4 provided complete tumor protection, with no detectable tumor growth observed for up to 30 days post-inoculation (Fig. 6, C and D). In contrast, 60% of mice treated with SM102 exhibited notable tumor progression, albeit at a slightly slower rate compared to the PBS-treated group.

To further elucidate the immunological mechanisms, antigen-specific immune responses were characterized. Flow cytometry conducted 72 hours postvaccination revealed that G-LNPs significantly up-regulated the presentation of the SIINFEKL peptide (derived from OVA) on MHC-I molecules, demonstrating superior antigen presentation compared to SM102 (Fig. 6, E and F). Furthermore, G-LNP formulations induced a higher proportion of CD86^+^ cells, a marker of APC activation, relative to SM102 (Fig. 6G). CD86 is critical for T cell costimulation, facilitating their activation and proliferation. The increased CD86 expression and superior antigen presentation observed with G-LNPs highlight their potent immunostimulatory potential. Accordingly, T cell activation was significantly elevated in G-LNP–treated mice. Using CD69 as a T cell activation marker, we found that after 24 hours of G-LNP treatment, GL5-3 activated more than 48% of CD4^+^ and 40% of CD8^+^ T cells, whereas SM102 activated only 5% of CD4^+^ and 7% of CD8^+^ T cells. GL5-4 achieved even greater activation, with 58% of CD4^+^ and 47% of CD8^+^ T cells activated, representing more than 10-fold and 6-fold improvements, respectively, compared to SM102 (Fig. 6H). We next investigated the effects of DC-mediated T cell activation by coculturing OVA-mRNA-LNP–transfected BMDCs (bone marrow–derived dendritic cells) with OVA-specific T cells. Although the overall levels of T cell proliferation were comparable between the G-LNP and SM102 treatment groups, a significantly higher proportion of T cells with >2 divisions were observed in the G-LNP groups (fig. S14).

The ability of G-LNPs to establish long-lasting immune memory was also investigated. Vaccinated mice were rechallenged with a higher dose of E.G7-OVA cells (1 × 10^6^) 30 days after the initial tumor inoculation (Fig. 6I). Impressively, these mice displayed complete resistance to tumor growth during the 30-day observation period (Fig. 5, J and K), suggesting robust and durable protective immunity. Flow cytometry analyses of spleens from rechallenged mice revealed noteworthy shifts in T cell subsets. Specifically, GL5-3 and GL5-4 promoted the differentiation of memory T cell populations, including effector memory T cells (T_em_) and central memory T cells (T_cm_). While the extent of changes varied, there were notable reductions in CD8^+^ T_cm_ and CD4^+^ T_em_ cells (Fig. 6, L and M). Effector memory T cells are known to mediate rapid, antigen-specific responses upon re-exposure, while central memory T cells ensure long-term immune surveillance and adaptability (*77*). This balance between T_em_ and T_cm_ subsets suggests that G-LNPs are capable of inducing a robust, durable, and adaptable immune memory.

To address their therapeutic potential, G-LNPs were evaluated in a model simulating established tumor conditions. Mice bearing subcutaneous tumors were treated with OVA mRNA formulated in either guanidine-based or amine-based LNPs on days 10, 14, and 18. Both formulations GL5-3 and GL5-4 notably inhibited tumor growth and prolonged survival compared to controls (fig. S11). These results confirm the applicability of guanidine-based LNPs not only as prophylactic cancer vaccines but also as therapeutic agents for targeting existing tumors. This efficacy likely stems from enhanced antigen expression and potent activation of CD8^+^ T cells, leading to long-lasting immune responses against tumors.

Last, we assessed the biocompatibility and safety profile of G-LNPs (figs. S16 to S18). Both in vitro and in vivo studies revealed minimal cytotoxicity associated with GL5-3 and GL5-4 formulations. Histological staining of major organs showed no observable tissue damage. Standard biomarkers of organ function (*78*)—including ALT (alanine aminotransferase), AST (aspartate aminotransferase) and ALB (albumin)—were measured for the heart, liver, and kidneys. Biomarker levels for GL5-treated mice were similar to those treated with SM102 across multiple dosages, indicating excellent biocompatibility.

## DISCUSSION

Recent advancements in mRNA-based therapeutics, along with other nucleic acid–based therapies, represent a momentous breakthrough in disease treatment. Inspired by the remarkable success of mRNA vaccines against infectious diseases, there has been a growing effort to leverage mRNA technology across diverse applications, with cancer immunotherapy emerging as a particularly promising focus. However, despite these advancements, the development of effective mRNA-based treatments has been largely impeded by challenges related to delivery systems. Conventional amine-based LNP platforms, which are widely used for mRNA delivery, face limitations in achieving efficient, and targeted delivery to immune-relevant organs, such as the spleen, which is essential for activating robust immune responses.

In this study, we addressed these limitations by developing a specific class of ionizable guanidine lipids, which enabled the creation of guanidine-based LNPs (G-LNPs) with unique and enhanced properties. Among these formulations, GL5-3, a cholesterol-free, three-component LNP, exhibited exceptional performance by efficiently delivering functional mRNA directly to the spleen. Furthermore, G-LNPs demonstrated superior immunotherapeutic efficacy compared to amine-LNPs, particularly through enhanced antigen presentation and T cell activation, which is essential for robust activation of immune pathways. Considering that GL5-4 and SM102 both delivered mRNA to the liver and spleen, the observed difference in T cell activation is remarkable. These results demonstrated that G-LNPs elicit substantially stronger antigen presentation and immune cell activation than amine-LNPs, which are pivotal for effective cancer immunotherapy.

The superior performance of G-LNP might originate from their preferential ability to activate helper T cells. In particularly, the activation of CD69 necessitates antigen presentation by APCs in the spleen, wherein the interaction between MHC molecules on APCs and the TCR initiates the CD3/interleukin-2 signaling pathway, culminating in the up-regulation of CD69. The population of activated CD4^+^ T cells in mice treated with G-LNP was over 10-fold greater than that in mice treated with amine-LNP. It is well-documented that APCs activate CD8^+^ T cells by presenting endogenous antigens through MHC class I molecules, which is regarded as the principal pathway of cellular immune activation for mRNA vaccines targeting APCs (*72*). Conversely, the activation of CD4^+^ T cells is mediated via the presentation of exogenous antigens by MHC class II molecules. We observed a sustained immune memory response against tumor cells lasting up to 50 days, likely attributable to CD4^+^ T cell–mediated humoral immunity. In addition, the activation of CD4^+^ T cells suggests that G-LNPs may enhance the phagocytic and antigen-processing capabilities of APCs, potentially leading to epitope spreading (*79*). A detailed investigation into the mechanisms of CD4^+^ T cell activation is needed. Meanwhile, our findings indicate that G-LNPs more effectively promote the proliferation of specific CD8^+^ T cell subsets compared to SM-102 LNPs. Given that different T cell subsets exhibit distinct characteristics, such as exhaustion or stem-like properties (*80*), the specific CD8^+^ T cells activated by G-LNP may contribute to the observed long-lasting immune memory. Therefore, further analysis of these subsets, with a focus on exhaustion and stemness markers, is crucial for elucidating the mechanisms of long-term immunity.

In summary, G-LNPs represent a distinct class of LNPs with enhanced antigen presentation and immune activation properties compared to widely used amine-LNPs. This achievement is further complemented by the ability of G-LNPs to establish durable immunological memory. Our findings underscore the unique properties of ionizable guanidine lipids in developing organ-targeted LNPs and open new avenues for advancing mRNA cancer vaccines.

## MATERIALS AND METHODS

All fatty acids were purchased from Macklin (Shanghai, China). Dulbecco’s modified Eagle’s medium (DMEM), RPMI 1640 medium, FluoroBrite DMEM, penicillin (10,000 U/ml), and streptomycin (10 mg/ml), Fetal bovine serum (FBS), trypsin-EDTA (0.25%), and PBS (1:250) were purchased from Gibco (California, USA). DOPE, 1,2-dimyristoyl-rac-glycero-3-methoxypolyethylene glycol-2000 (DMG–PEG 2000), cholesterol, 1,2-distearoyl-*sn*-glycero-3-phosphocholine, POPG, and POPC were supplied by Avanti Polar Lipids (Alabaster, USA). ANTS, DPX, thiazoyl blue tetrazolium bromide, and propidium iodide (PI) were purchased from Meryer (Shanghai, China). The Firefly Luciferase mRNA, Cy5 Firefly Luciferase mRNA, d-luciferin (potassium salt), and Luciferase Reporter Assay Kit were obtained from Vazyme (Nanjing, China). The OT-1 cells were a gift of P. Chen, and BMDCs were generated by the Zhiping Xu laboratory. The human cervical cancer cell line HeLa, human breast carci-noma cell line MDA-MB-231, and human astrocytoma cell line U251 were maintained in DMEM with 10% FBS, penicillin (100 U/ml), and streptomycin (100 μg/ml) at 37°C in a humidified atmosphere with 5% CO_2_. All animal experiments were approved by the Institutional Animal Care and Use Committee of Shenzhen Bay Laboratory (AELM202301 and AELM202401). Female Balc/b mice (6 to 8 weeks, 18 to 20 g) and C57BL/C mice (5 to 6 week, 18 to 20 g) were purchased from GemPharmatech Co. Ltd. (Nanjing, China).

### Preparation of LNP

GL-lipids were dissolved in dimethyl sulfoxide (50 mg/ml), DOPE, cholesterol, and DMG–PEG 2000 were dissolved in ethanol (10 mg/ml). The four components were thoroughly mixed according to the indicated ratios. The Luc-mRNA (encoding the firefly luciferase) was dissolved in nuclease-free water (1 mg/ml) and diluted with 10 mM citrate buffer (pH 4.0). For in vitro studies, the two solutions were mixed with pipettes (total lipids: mRNA = 1:3), followed immediately by pipetting up and down rapidly for 30 s. The resulting solutions were diluted with PBS and used immediately for cellular experiments. For in vivo studies, the LNPs were prepared through microfluidic mixing using INano L^+^ (Micro&Nano) and ultrafiltration with PBS. For preparing DiR-labeled LNPs, DiR dye was added into the lipid solution in ethanol at about 1% mol, respectively. LNPs were prepared through microfluidic mixing and dialyzed into PBS solution using a 10K MWCO Slide-A-Lyzer (Thermo Fisher Scientific).

### In vitro mRNA delivery

For lipid screening, LNPs were prepared as described with a fixed molar ratio of 50:10:38.5:1.5 for GL-lipids, DOPE, cholesterol, and DMG–PEG 2000, respectively. For formulation optimization, GL-lipids, DOPE, cholesterol, and DMG–PEG 2000 were divided into 32 groups, and LNPs were prepared as described according to the molar ratios shown in fig. S2 to S4. HeLa cells (1 × 10^4^) were seeded into 96-well plates overnight, and LNPs were added (150 ng mRNA per well) to the cells in DMEM (10% FBS). After 6 hours of incubation, the medium was removed and replaced with fresh medium to continue the incubation for 18 hours. The expression levels of luciferase were measured using the Luciferase Reporter Assay Kit in plate reader (Agilent BioTek Synergy H1).

### Cryo-EM

The Vitrobot IV was used for sample preparation for cryo-TEM. The humidity was maintained at 100% throughout all experiments, and the temperature was set at 10°C. A 300-mesh copper grids coated with carbon film (Quantifoil R 1.2/1.3 300 Mesh, Cu) were subjected to glow discharge using a Pelco EasiGlow Glow Discharge unit to render the carbon film hydrophilic. Subsequently, 3-μl aliquots of the sample were pipetted onto each grid before plunging. After an adsorption time of 10 s, the grid was manually blotted using Ted Pella filter paper for 2.5 s before being plunged into liquid ethane cooled by liquid nitrogen. Frozen grids were then stored in liquid nitrogen until required. The samples were examined using a Tundra operating at a voltage of 100 kV and an electron dose of 38 e^–^/Å for all imaging purposes. Images were captured using a CETA F camera.

### ANTS release assay

POPC/POPG (3:1) solution was prepared at 10 mg/ml in 5 mM Hepes buffer (pH 7.4). ANTS and DPX were added to a final concentration of 50 mM. The solution is vortexed and subjected to five freeze/thaw cycles. LUVs were produced via membrane extrusion 10 times using Mini-Extrude (Avanti Polar Lipids), and the excess dyes were removed with a desalting column (Cytiva PD-10). To measure the membrane permeability, 100 μl of LUV solution was added per well into a 96-well plate, and LNPs were added at a concentration of 0 to 2.5 μg (total lipid per well). After 5 min of incubation in the dark at room temperature, the fluorescence intensity was recorded using a plate reader (Agilent BioTek Synergy H1) at excitation and emission wavelengths of 350 and 520 nm, respectively.

### Hemolysis experiment

Mouse red blood cells (RBCs) were isolated and washed three times with 1× PBS by centrifugation at 3000 rpm for 5 min. The RBCs were diluted to 5% RBC solution in PBS (pH 7.4) or citrate buffer (pH 5.5). A 100 μl of RBC solution and LNP containing 50 ng of mRNA were added to a 96-well plate and incubate at 37°C for 1 hour. After centrifugation at 3000 rpm for 5 min, the supernatant was collected, and the absorbance was measured at 540 nm with a plate reader. Positive and negative controls were carried out with 0.2% Triton and 1× PBS.

### Confocal microscopy imaging

A total of 1 × 10^4^ HeLa cells were seeded in a μ-Slide 8 Well plate (ibidi) and incubated overnight. The cells were incubated with GL5 LNPs (loaded with Cy5-mRNA) for 3, 6, 12, and 24 hours. Hoechst 33342 and LysoTracker Green DND-26 were then added to stain the nucleus and lysosomes, respectively. After rinsing the cells three times with PBS, Fluoro Brite DMEM was added, and images were taken with a spinning disk confocal microscope (Olympus SpinSR). The images were processed with Fiji.

### In vivo mRNA delivery

Intravenous injection was applied for the in vivo experiments unless otherwise noted. Each female Balb/c mice (*n* = 3) was treated with Luc mRNA-loaded LNPs at an mRNA does of 0.2 mg/kg. After 6 hours, the mice were anesthetized with isoflurane and injected with 100 μl of d-luciferin (potassium salt) (30 mg/ml). After 10 min, the mice were imaged using an IVIS Spectrum (PerkinElmer). Using a similar approach, mice were executed 5 to 10 min after injection of d-luciferin and organs (heart, liver, spleen, lung, and kidney) were immediately removed and imaged. The average radiance in isolated organs was quantified with Living Image software.

### Splenic cell sorting

Cy5-Luc-mRNA was used to examine the biodistribution of LNPs in the spleen. Each female Balb/c mice (*n* = 3) was intravenously injected with Cy5-Luc-mRNA/LNPs at an mRNA does of 0.5 mg/kg. After 6 hours, the mice were euthanized, and spleens were harvested. Spleens were placed in RPMI 1640 medium and homogenized using a 200-mesh cell strainer and centrifuged (300*g*, 5 min) at 4°C. The supernatant was removed, and the RBC lysis buffer was added for 5 min at room temperature. PBS buffer was added, and the supernatant was discarded after centrifugation (300*g*, 5 min). The pellet was resuspended to obtain single-cell suspension. The cells were incubated with indicated antibodies for 60 min in the dark at 4°C for flow cytometry analysis using a LSR Fortessa SORP (BD Biosciences). The following antibodies were used: Alexa Fluor 700 anti-mouse CD45 antibody (immune cells), polyethylene (PE)/cyanine7 anti-mouse CD3 antibody (T cells), BV421 anti-mouse/human CD45R/B220 (B cells), fluorescein isothiocyanate (FITC) anti-mouse/human CD11b antibody, and BV 605 anti-mouse CD11c antibody (DCs).

### Liver cell sorting

Cy5-Luc-mRNA was used to examine the biodistribution of LNPs in the liver. Each female Balb/c mice (*n* = 3) was intravenously injected with Cy5-Luc-mRNA/LNPs at an mRNA does of 0.5 mg/kg. After 6 hours, the mice were euthanized and livers were harvested. Metal ions were removed by retrograde perfusion through the inferior vena cava to the portal vein using perfusion buffer (25 mM Hepes and 5 mM EDTA in Hanks’ balanced salt solution), followed by perfusion using digest buffer (25 mM Hepes, 0.02% collagenase IV, and 0.002% deoxyribonuclease 1) to disperse the cells. The supernatant was obtained through centrifugation at 500*g* for 5 min to isolate liver nonparenchymal cells. These cells were subsequently resuspended in a red blood cell lysis buffer and incubated for 5 min. Following incubation, the cells were washed twice with 1× PBS and resuspended in 100 μl of 1× PBS. The cell suspension was then incubated with antibodies, including PE-labeled anti-CD45 (BioLegend, 103106), BV711-labeled anti-F4/80 (BioLegend, 123147), and FITC-labeled anti-CD146 (BioLegend, 134705). The analysis of the cell suspension was conducted using a multicolor analytical flow cytometer (LSR Fortessa, BD Biosciences). Single liver nonparenchymal cells were identified on the basis of the following markers: LSECs as CD146^+^ CD45^−^ and Kupffer cells as CD146^−^ CD45^+^ F4/80^+^.

### In vitro T cell proliferation study

For in vitro antigen-specific T cell proliferation assay, 0.5 × 10^6^ BMDCs were seeded in a 24-well plate. Subsequently, OVA-mRNA-LNP (1 μg/ml mRNA) were added and incubated with BMDCs for 16 hours. Following the transfection, BMDCs were cocultured with 2 × 10^6^ carboxyfluorescein diacetate succinimidyl ester (CFSE)–labeled OT-1 T cells for a duration of 3 days. Upon completion of the coculture, cells were harvested and stained with Pacific Blue–conjugated CD8a antibody. Flow cytometry is then performed to evaluate T cell proliferation, as indicated by the dilution of CFSE.

### Mice vaccination

C57BL/C mice aged 5 to 6 weeks were vaccinated through intravenous injection with different LNPs containing 10 μg of OVA mRNA on days 0, 7, and 14 with a total of three doses. On day 21, 5 × 10^5^ E.G7-OVA cells were subcutaneously injected. For the tumor rechallenge experiment, 1 × 10^6^ E.G7-OVA cells were subcutaneously injected on day 50. Tumor growth was measured three times a week using a digital caliper, and the volume was calculated as 0.5 × length × width × width. When the tumor volume reached 2000 mm^3^, the mice were euthanized.

### Antigen presentation and T cell analysis

Spleens were placed in RPMI 1640 medium and homogenized using a 200-mesh cell strainer and centrifuged (300*g*, 5 min) at 4°C. The supernatant was removed, and RBC lysis buffer was added for 5 min at room temperature. PBS buffer was added, and the supernatant was discarded after centrifugation (300*g*, 5 min). The pellet was resuspended to obtain single-cell suspension. The cells were incubated with indicated antibodies for 60 min in the dark at 4°C for flow cytometry analysis using a LSR Fortessa SORP (BD Biosciences). The following antibodies were used: Alexa Fluor 700 anti-mouse CD45 antibody, PE/cyanine7 anti-mouse CD3 antibody, PacificBlue anti-mouse CD8a antibody, APC anti-mouse CD4 antibody, PerCP anti-mouse CD44 antibody, FITC anti-mouse CD62L antibody, PE/Cy5 anti-mouse CD69 antibody, PE anti-mouse CD103 antibody, PE/cyanine7 anti-mouse H-2Kb bound to SIINFEKL, and FITC anti-mouse CD86 antibody.
