# Supplementary material for: Ionizable guanidine-based lipid nanoparticle for targeted mRNA delivery and cancer immunotherapy
Source: Sci Adv. 2025 Oct 24;11(43):eadx5970. doi: 10.1126/sciadv.adx5970 (PMC12551712; doi:10.1126/sciadv.adx5970)
Supplement: Supplementary file 1 — Supplementary Text Figs. S1 to S18 Tables S1 to S3 [file sciadv.adx5970_sm.pdf]

Science Advances
AAAS

Supplementary Materials for

Ionizable guanidine-based lipid nanoparticle for targeted mRNA delivery and cancer immunotherapy

He Zhang *et al.*

Corresponding author: Mao Li, limao@szbl.ac.cn

Sci. Adv. **11**, eadx5970 (2025)
DOI: 10.1126/sciadv.adx5970

This PDF file includes:

Supplementary Text
Figs. S1 to S18
Tables S1 to S3

Supplementary Text

In vitro GFP mRNA delivery

LNPs encapsulating GFP-mRNA were prepared as described with GL5-lipid, DOPE, and DMG-PEG2000 in a molar ratio of 40:40:0.75 with indicated amount of mRNA. 5×10^4 HeLa cells were seeded into 24-well plates, and LNP at a concentration of 250ng GFP-mRNA was added to each well. After 16h of incubation, the medium was removed and replaced with fresh complete medium to continue the incubation for 24 h. Cells were then washed with PBS (3 × 250μL) and treated with trypsin-EDTA (100 μL) for 5 min at 37°C. Subsequently PBS (300 μL, 4 ºC) was added, cells were gently suspended and centrifuged at 1500 rpm for 5 min. The supernatant was carefully removed and the pellet resuspended in a solution of 1.5 μM propidium iodide (PI) and 2 mM ethylenediaminetetraacetic acid (EDTA) in PBS (400 μL) at 4 ºC. The cell suspensions were placed in FACS tubes and kept on ice prior to analysis on a BDLSFRortessa flow cytometer. Events corresponding to cellular debris were removed by gating on forward and side scatter. Each sample was run in triplicate (3 × 10,000 events) at the day of measurement and the entire experiment was repeated at least three times.

Dynamic light scattering

The size and zeta potentials of LNPs were measured using dynamic light scattering (Zetasizer Pro, Malvern Panalytical). LNP was diluted to 0.5mg/ml in PBS and tested for particle size and zeta potential at 25°C. Each sample was examined three times. Diameters are reported as intensity mean peak average.

pKa determination

The pK_a of the LNPs was determined using a TNS assay, which is widely used to evaluate the apparent pK_a of LNPs. 2 µL LNPs (6mM) were mixed with 106µL 10mM (HEPES, MES, Citrate) containing 130mM NaCl buffers from pH 3 to 11 to a 96-well plate. Afterwards, 12µL 100µM TNS solution was added to each well followed by incubation for 15min. Fluorescence intensity was recorded using a plate reader (Agilent BioTek Synergy H1) at excitation and emission wavelengths of 321nm and 445nm. The data was analyzed with GraphPad Prism.

Luciferase expression in isolated organs

For in vivo mRNA delivery, each female Balb/c mice (n = 3) was i.v. injected with Fluc mRNA-loaded LNPs at an mRNA does of 0.2mg/kg. After 6 h, the mice were anesthetized with isoflurane and injected with 100µL D-Luciferin (Potassium Salt) (30 mg/mL). Mice were

sacrificed 5-10 minutes later and organs were collected and added to 500μL PBS for homogenization using a High-Throughput Tissue Grinder (Scientz). The evaluation of luciferase expression in supernatants was performed using a Luciferase Reporter Assay Kit.

Tumor inhibition experiment

C57BL/C mice aged 5-6 week were injected subcutaneously with 5×10^5 E.G7-OVA cells. LNP-OVA mRNAs were i.v. injected when the tumor size reached 50-100 mm^3 (on the 10th day after tumor inoculation). Mice were treated with different LNP formulations containing 10μg OVA mRNA and a total of three doses were given. Tumor growth was measured three times a week using a digital caliper, and the volume was calculated as 0.5×length×width×width. When the tumor volume reached 2,000 mm^3, the mice were euthanized.

MTT assay

1×10^4 HeLa cells were seeded into 96-well plates, and LNP at a concentration of 500ng mRNA was added to each well. After 16h of incubation, the medium was removed and replaced with fresh complete medium to continue the incubation for 12h. The medium was removed and the cells were washed twice with PBS, 50μL MTT solution (5mg/mL) and 50μL fresh DMEM were added to each well, incubated for 2h and then the medium was removed. Afterwards, 100μL DMSO was added to each well and the absorbance was recorded using a plate reader (Agilent BioTek Synergy H1) at 490 nm.

Supplementary Results

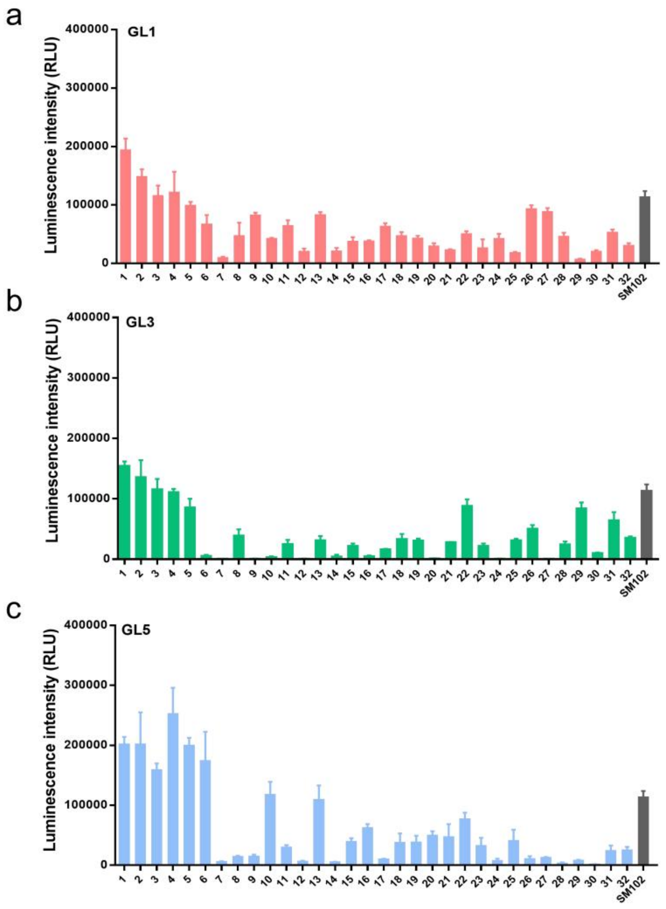

Figure S1. Orthogonal Screening of LNPs. Luciferase mRNA expression levels were generated from the in vitro mRNA delivery experiments of GL1 (a), GL3 (b) and GL5 (c) in HeLa cells (150 ng mRNA per well). SM102 was applied as a positive control.

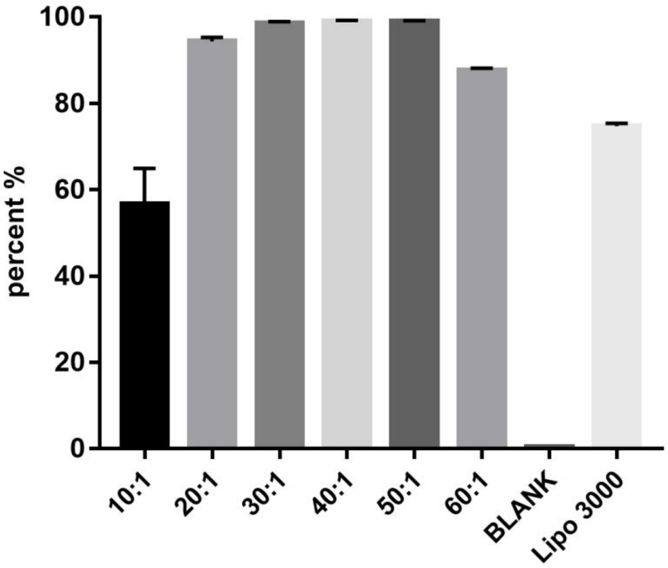

Figure S2. GFP mRNA delivery efficiency at different mass ratios of GL5-LNP and mRNA. 5×10^4 HeLa cells were treated with various LNPs at a dose of 250ng GFP-mRNA/well for 16h. The expression level of GFP was measured using flow cytometry and analyzed with Flowjo.

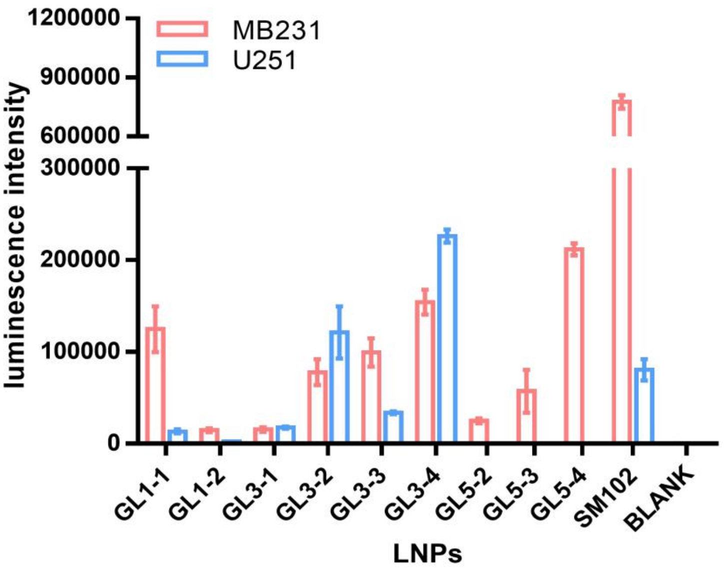

Figure S3. mRNA delivery efficiency of screened LNPs in MB231 and U251 cells. 1×10^4 Cells were treated with various LNPs at a dose of 150ng Luc-mRNA/well for 6h. The expression level of luciferase was measured using the Luciferase Reporter Assay Kit.

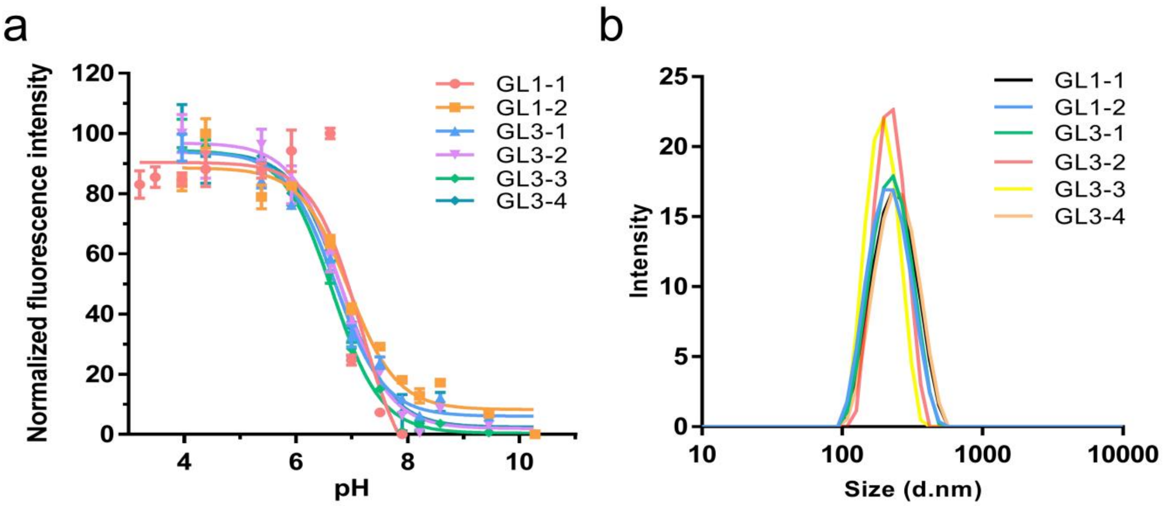

Figure S4. Particle size and pKa of LNPs. (a)TNS fluorescence assays and (b) DLS size distribution profiles for the selected LNPs

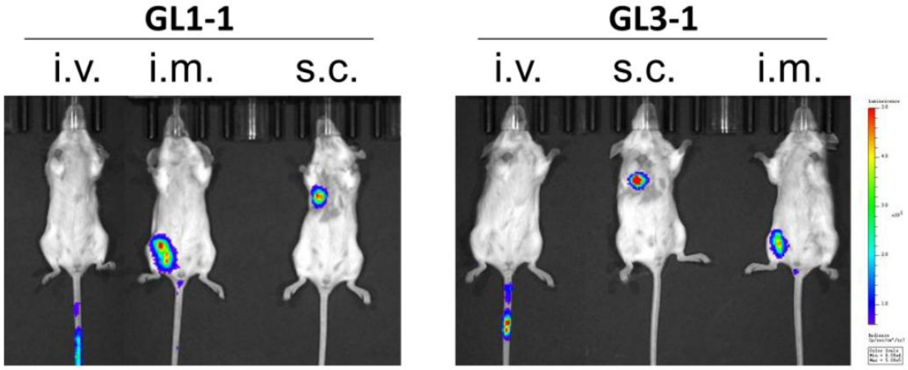

Figure S5. In vivo mRNA delivery of GL1-1 and GL3-1. Female Balb/c mice were i.m. and s.c. injected with Fluc mRNA-loaded LNPs at an mRNA does of 0.2mg/kg. After 6h, the mice were imaged using an IVIS Spectrum (Perkin Elmer).

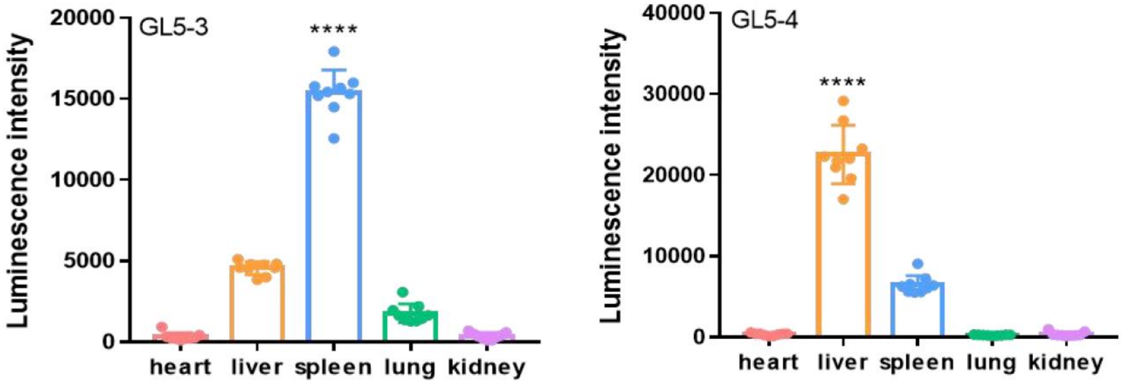

Figure S6. **Luciferase expression levels of GL5-3 and GL5-4.** Female Balb/c mice received intravenous injections of GL5-3 and GL5-4 containing mRNA at a dose of 0.2 mg/kg. 6 hours later, luciferase expression levels in organs were measured using a luciferase assay kit.

a

GL5	lipid	DOPE	PEG
1A	32.80	65.70	1.5
1B	49.25	49.25	1.5
1C	70.40	28.10	1.5
1D	89.50	9.00	1.5
1E	93.80	4.70	1.5

b

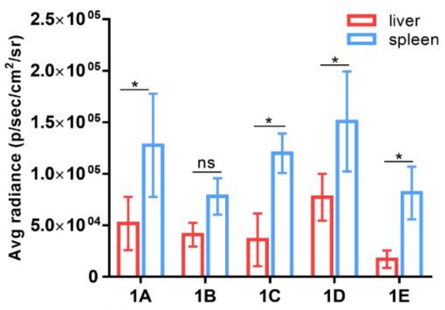

c

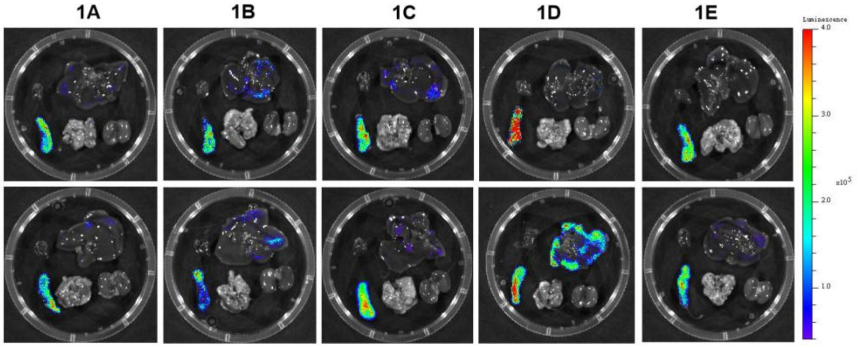

Figure S7. Effect of GL5 lipid to DOPE ratio variation. (a) Table of the formulations for the GL5 LNPs with varying ratios between the GL5-lipid and DOPE. (b) Average radiances in the spleen and liver for the mice treated with 3-components GL5 LNPs. (c) In vivo biodistribution of the mRNA delivery properties of the 3-components GL5 LNPs.

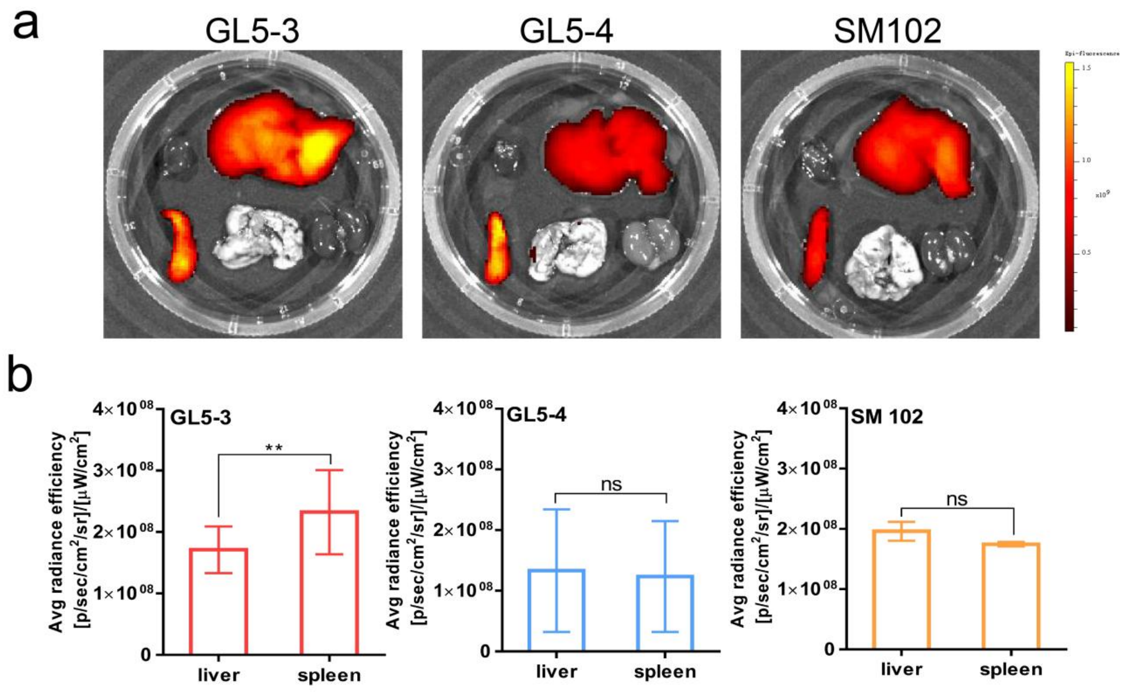

Figure S8. LNP accumulation in vivo. In vivo biodistribution (a) and quantification (b) of different LNPs labelled with Dir. Female Balb/c mice were i.v. injected with Dir-labled LNPs at an mRNA does of 0.2mg/kg. After 6 h, the mice were imaged using an IVIS Spectrum (Perkin Elmer).

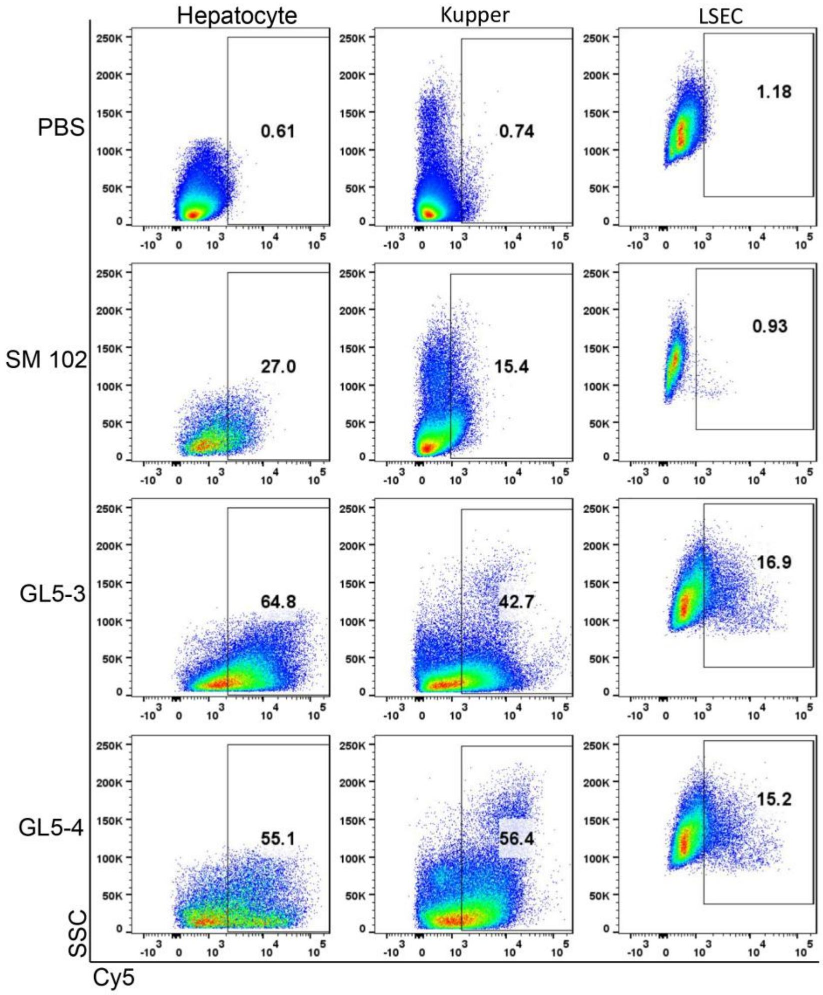

Figure S9. The FACS gating strategy for analysis of Cy5+ expression in liver cells is described. CD45+ was used to define immune cells, CD45+ and F4/80 were used for Kupffer cells, CD146 and Retinoid were used for LSEC cells and hepatocyte cells. Gates for Cy5+ in cell types were drawn based on PBS injected control mice. Balb/c mice were injected with Cy5-mRNA loaded LNPs formulations and Cy5+ in given cell types was detected after 6 hours (n=3).

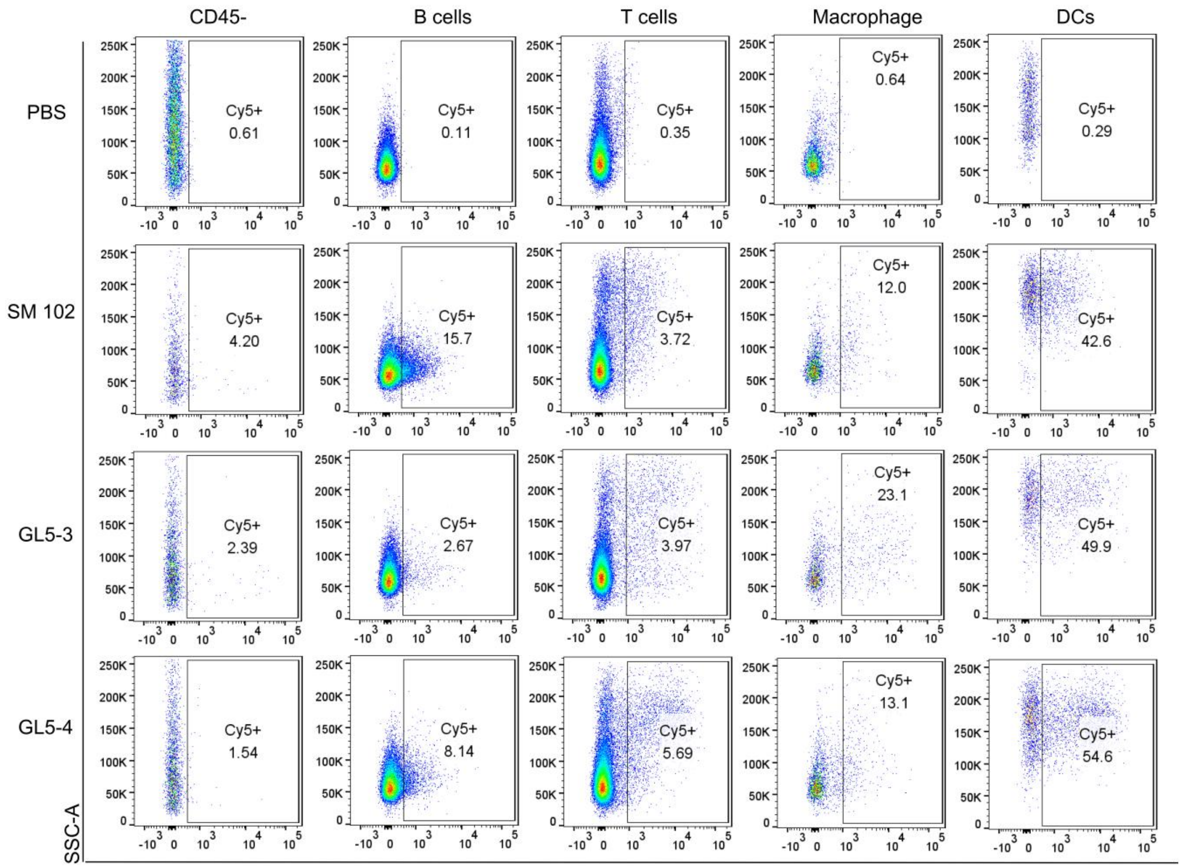

Figure S10. The FACS gating strategy for analysis of Cy5+ expression in spleen cells is described. CD45+ was used to define immune cells, CD45+ and CD3+ were used to define T cells, CD45+ and CD45R/B220+ were used to define B cells, F4/80+ was used to define macrophages cells, CD11b+ and CD11c+ were used to define DCs. Gates for Cy5+ in cell types were drawn based on PBS injected control mice. Balb/c mice were injected with Cy5-mRNA loaded LNPs formulations and Cy5+ in given cell types was detected by flow after 6 hours (n=3).

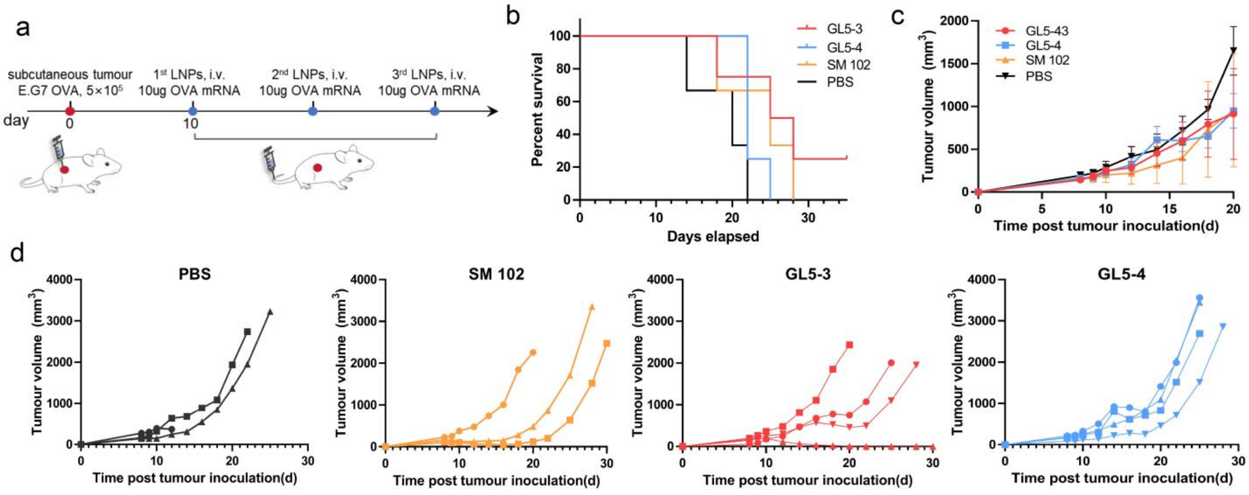

Figure S11. GL5 LNPs induce potent tumor-preventive immune responses.(a) Schematic representation of the tumor inhibition model in C57BL/6 mice. Mice were injected subcutaneously with EG7-OVA cells, followed by three i.v. injections of mOVA-loaded GL5-3, GL5-4, SM102 (10μg mOVA per injection) and PBS. (b) Survival curves and (c) tumor volumes of mice treated with LNPs. (d) Tumor volumes of individual mice treated with LNP-OVA mRNA.

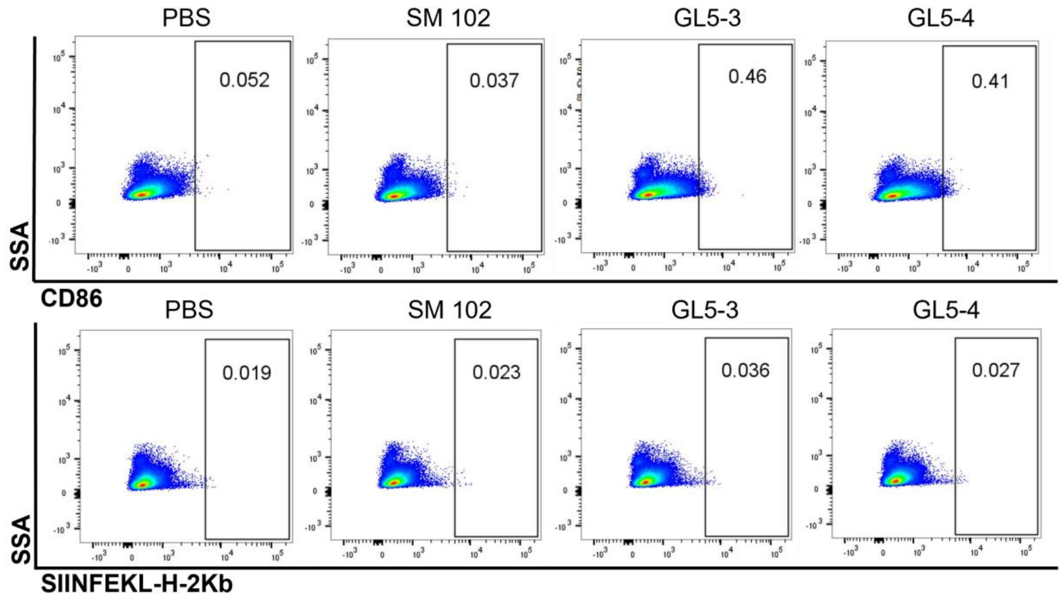

Figure S12. Representative flow cytometry plots for analysis of SIINFEKL-H-2Kb^{+} and CD86^{+} cells on day 3 post administration. C57BL/6 mice were given one i.v. injection of PBS, GL5-3, GL5-4 or SM 102 LNPs loaded with mOVA. Mice were sacrificed three days after the injection, and their spleen were isolated for analysis. Cells positive for SIINFEKL-H-2Kb and CD86 are shown.

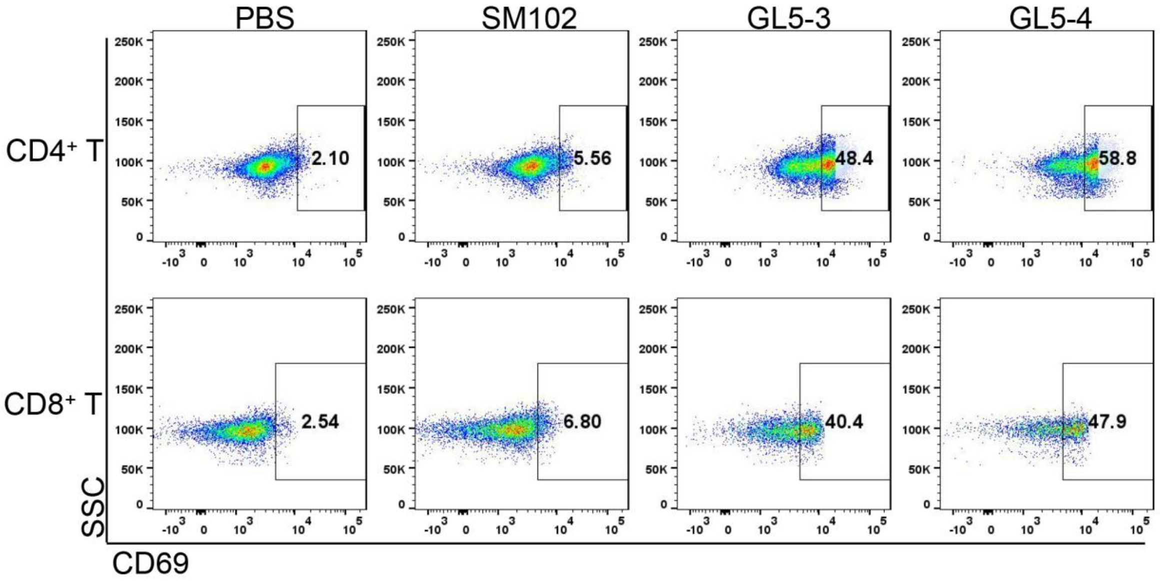

Figure S13. The FACS gating strategy for analysis of activated T cells in spleen. C57BL/6 mice were given one i.v. injection of PBS, GL5-3, GL5-4 or SM 102 LNPs loaded with mOVA. Mice were sacrificed three days after the injection, and their spleen were isolated for analysis. Cells positive for CD69 are shown.

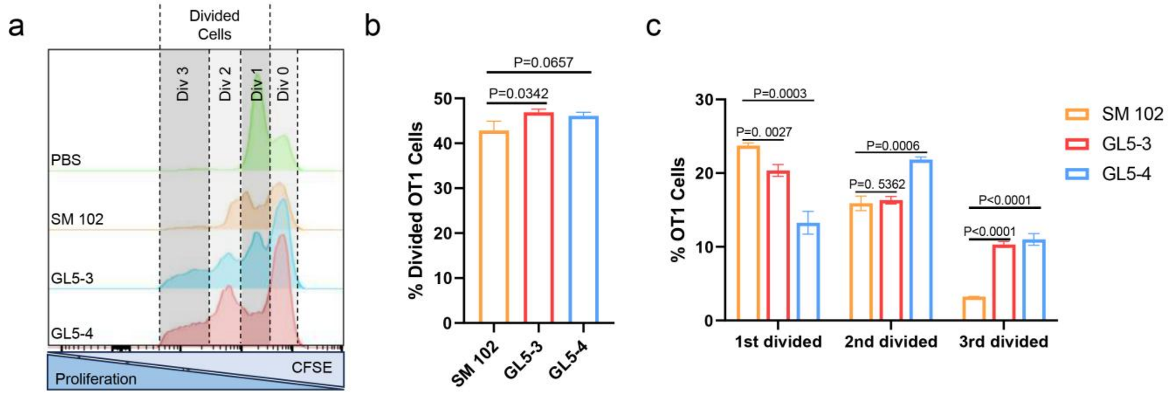

Figure S14. Analyzing T cell proliferation through CFSE dilution assay. Flow cytometric analysis of CFSE-labeled OT1 CD8+ T cell proliferation cultured with different OVA-mRNA-LNP induced BMDC for 3 days. Fraction of OT-1 which underwent 0, 1, 2, 3 cell divisions of proliferation after co-culture(a, b, c)

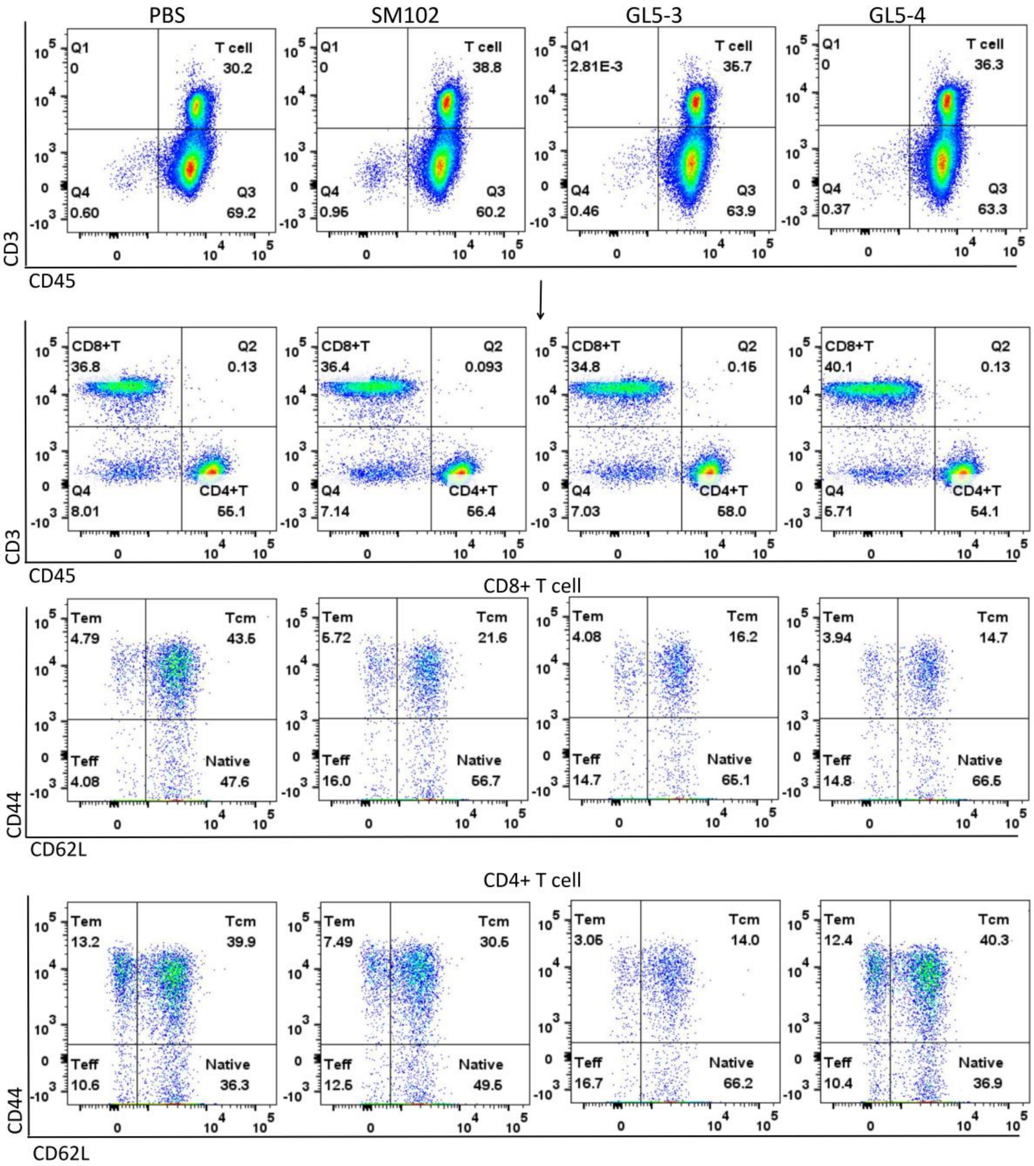

Figure S15.The FACS gating strategy for analysis of memory T cells in spleen. Vaccinated mice were rechallenged with a higher dose of E.G7-OVA cells (1×10^6) 30 days after the initial tumor inoculation. Spleens of mice were analyzed by flow cytometry for CD8+ and CD4+ memory T cells.

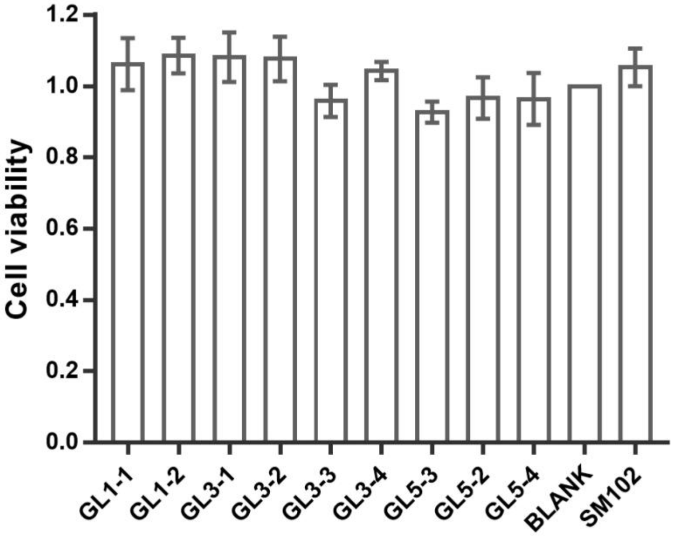

Figure S16. MTT assay for LNPs. 1×10^4 HeLa cells were treated with various LNPs at a dose of 500 ng Luc-mRNA/well for 16 h.

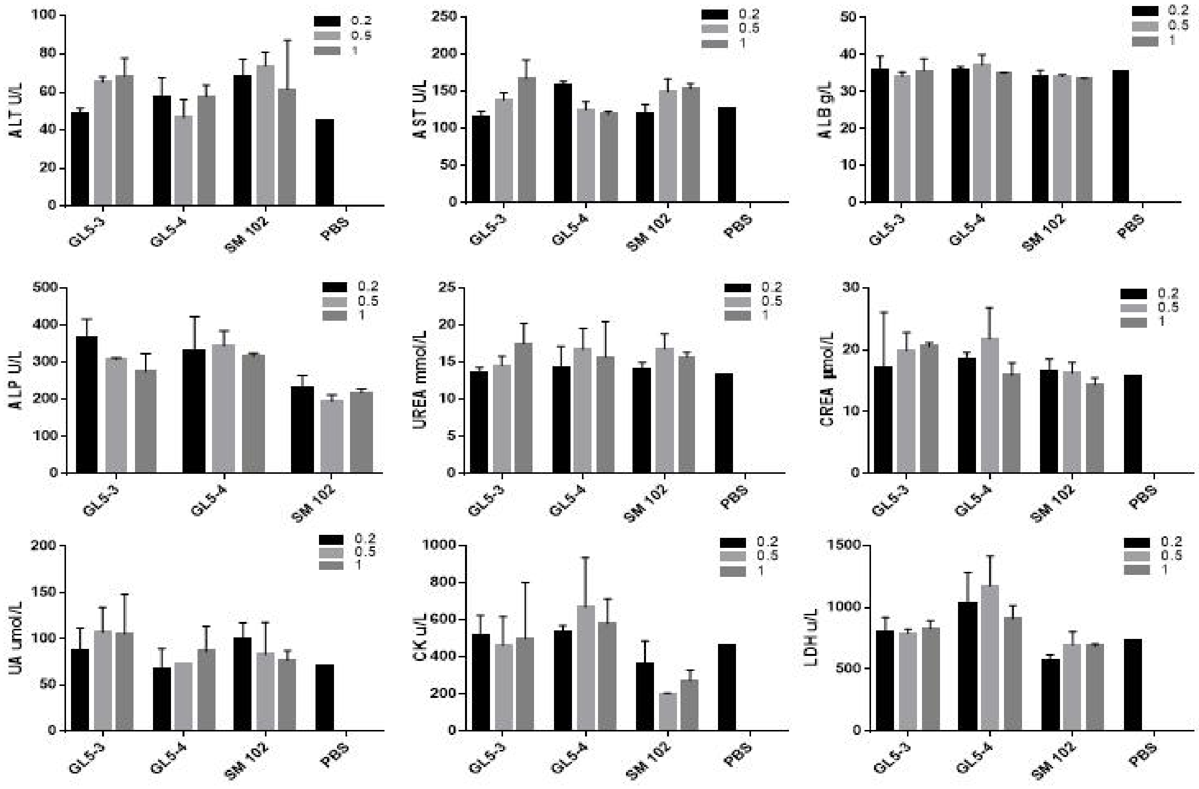

Figure S17. **Serum biochemical index of mice treated with LNPs GL5-3, GL5-4 and SM 102.** Serum was collected 24 hours after treatment with 0.2, 0.5, 1.0 mg/kg Luc mRNA-loaded LNPs.

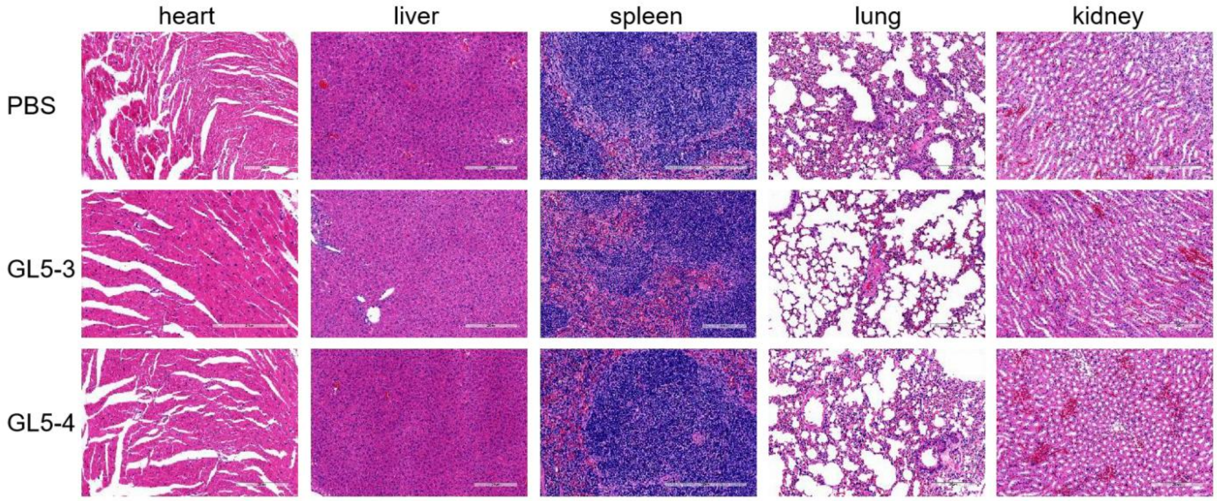

Figure S18. **In vivo safety evaluation of GL5-LNPs.** Organs were collected 24 hours after treatment with Luc mRNA-loaded LNPs (1mg/kg). Organs were embedded in paraffin and cut into 5 μm sections which were stained with hematoxylin and eosin (HE). The sections were observed using Digital Pathology Scanner (Leica Aperio VERSA 200).

entry	GL1	DOPE	cholesterol	PEG 2000	entry	GL1	DOPE	cholesterol	PEG 2000
**1**	50	7.5	33.5	0.6	**17**	50	20	48.5	3
**2**	40	15	33.5	1	**18**	50	15	43.5	2
**3**	40	2.5	48.5	3	**19**	50	10	38.5	1
**4**	60	1.25	18.5	0.75	**20**	60	10	48.5	6
**5**	50	5	48.5	0.75	**21**	45	20	43.5	1
**6**	50	10	38.5	1.5	**22**	45	15	48.5	0.5
**7**	50	2.5	18.5	6	**23**	45	10	33.5	3
**8**	50	1.25	28.5	3	**24**	45	7.5	38.5	2
**9**	40	10	28.5	0.75	**25**	40	20	38.5	0.5
**10**	40	5	18.5	1.5	**26**	60	5	38.5	3
**11**	60	2.5	28.5	15	**27**	40	10	48.5	2
**12**	40	1.25	38.5	6	**28**	40	7.5	43.5	3
**13**	30	10	18.5	3	**29**	35	20	33.2	2
**14**	30	5	28.5	6	**30**	35	15	38.5	3
**15**	30	2.5	38.5	0.75	**31**	35	10	43.5	0.5
**16**	30	1.25	48.5	1.5	**32**	35	7.5	48.5	1

Table S1. Formulation screening of GL1-LNP.

**entry**	GL3	DOPE	cholesterol	PEG 2000	**entry**	GL3	DOPE	cholesterol	PEG 2000
**1**	55	30	12.5	0.75	**17**	35	20	12.5	1.5
**2**	45	30	18.5	0.5	**18**	60	2.5	18.5	0.75
**3**	55	20	0	0.5	**19**	45	40	12.5	2
**4**	40	40	0	0.75	**20**	50	10	38.5	5
**5**	40	10	18.5	1.5	**21**	35	30	0	2
**6**	40	1.25	38.5	0.75	**22**	40	10	12.5	0.5
**7**	50	1.25	18.5	3	**23**	45	10	0	1.5
**8**	60	5	38.5	1.5	**24**	30	2.5	38.5	3
**9**	30	1.25	48.5	1.5	**25**	35	10	18.5	0.75
**10**	40	5	28.5	3	**26**	40	30	6.5	1.5
**11**	50	5	48.5	0.75	**27**	30	5	18.5	5
**12**	60	1.25	28.5	6	**28**	55	10	6.5	2
**13**	30	10	28.5	0.75	**29**	35	40	6.5	0.5
**14**	40	2.5	48.5	6	**30**	40	20	18.5	2
**15**	50	2.5	28.5	1.5	**31**	45	20	6.5	0.75
**16**	60	10	48.5	3	**32**	55	40	18.5	1.5

Table S2. Formulation screening of GL3-LNP.

entry	GL5	DOPE	cholesterol	PEG 2000	entry	GL5	DOPE	cholesterol	PEG 2000
**1**	45	7.5	18.5	0.5	**17**	50	10	18.5	3
**2**	40	5	18.5	1.5	**18**	50	7.5	10.5	1.5
**3**	50	2.5	0	0.5	**19**	50	5	5.5	0.75
**4**	50	10	38.5	1.5	**20**	60	2.5	28.5	1.5
**5**	40	10	28.5	0.75	**21**	45	10	10.5	0.75
**6**	50	5	48.5	0.75	**22**	60	10	48.5	6
**7**	50	2.5	18.5	6	**23**	45	5	0	3
**8**	50	1.25	28.5	3	**24**	45	2.5	5.5	1.5
**9**	60	1.25	18.5	0.75	**25**	40	10	5.5	0.5
**10**	60	5	38.5	1.5	**26**	40	7.5	0	0.75
**11**	40	2.5	48.5	3	**27**	40	5	18.5	1.5
**12**	40	1.25	38.5	6	**28**	40	2.5	10.5	3
**13**	30	10	18.5	3	**29**	35	10	0	1.5
**14**	30	5	28.5	6	**30**	35	7.5	5.5	3
**15**	30	2.5	38.5	0.75	**31**	35	5	10.5	0.5
16	30	1.25	48.5	1.5	32	35	2.5	18.5	0.75

Table S3. Formulation screening of GL5-LNP.

Synthesis of GL-lipids

Synthesis of **GCP head**

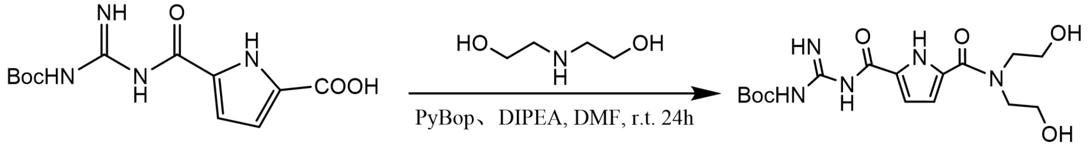

GCP(Boc) **GCP(Boc)-OH**

GCP(Boc)-OH (5g, 16.9mmol), diethanolamine (1.65ml, 1.8g, 16.9mmol), benzotriazole-1-yl-oxytripyrrolidinophosphonium hexafluorophosphate (PyBop) (26.3g, 50.05mmol) and N,N-Diisopropylethylamine (DIPEA) (17.45ml, 29g, 101.4mmol) were dissolved in 100ml DMF, and the reaction mixture was stirred for 24 hours at room temperature. The reaction mixture was extracted with water, DCM and the solvent was removed under reduced pressure. The residue was subjected to column chromatography (eluent: DCM : MeOH = 20 : 1,Rf=0.4) on silica gel to give **GCP head** as a yellow oil.

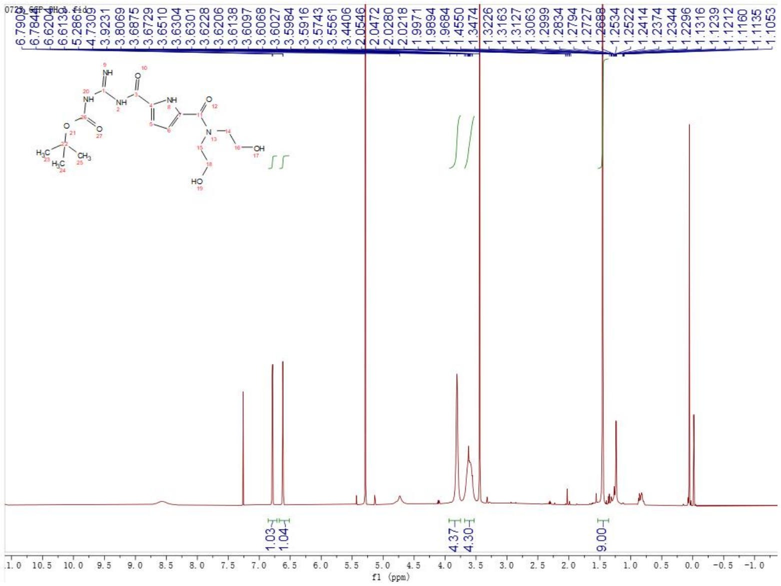

^{1}H NMR (600 MHz, Chloroform-*d*) δ 6.79 (d, J = 3.9 Hz, 1H), 6.62 (d, J = 3.9 Hz, 1H), 3.81 (s, 4H), 3.68 – 3.53 (m, 4H), 1.46 (s, 9H).

MS (LC-MS): m/z calculated for [C16H25N5O6]$^+$: 384.1877; found [M+H]$^+$:384.1878

General Procedure for the synthesis of **GL-lipids**

GCP head (100mg,0.295mmol), **Lipid Tails (L1-9)** (0.354mmol), 4-Dimethylaminopyridine (DMAP) (7.2mg, 0.059 mmol) and 1-(3-Dimethylaminopropyl)-3-

ethylcarbodiimide hydrochloride (EDC·HCl) (54.29m g, 0.354 mmol) were dissolved in 10ml dry DCM, and the reaction mixture was stirred at room temperature for 24 hours. The reaction mixture was washed with water, dried over Na_2SO_4 and the solvent was removed under reduced pressure. The residue was subjected to column chromatography on silica gel (eluent: DCM:MeOH=20:1, Rf=0.3) to afford the protected **GL lipid** as a clear oil. The product was then dissolved in 5 ml of TFA and stirred at room temperature for 2 h. Then the solvent was removed under reduced pressure and followed by lyophilization to provide **GL-lipid** as a white powder.

GL1:

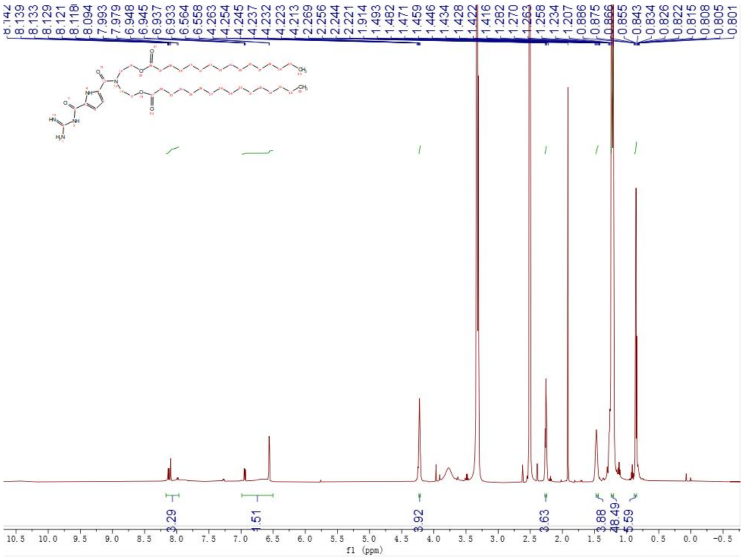

^{1}H NMR (600 MHz, DMSO-d_6) δ 8.17 – 7.97 (m, 3H), 6.99 – 6.50 (m, 2H), 4.22 (d, J = 5.8 Hz, 4H), 2.26 (d, J = 7.4 Hz, 4H), 1.47 (d, J = 7.2 Hz, 4H), 1.22 (d, J = 16.5 Hz, 48H), 0.86 (d, J = 6.7 Hz, 6H).

MS (LC-MS): m/z calculated for $[C43H77N5O6]^+$: 760.5947; found $[M+H]^+$:760.5949

GL2:

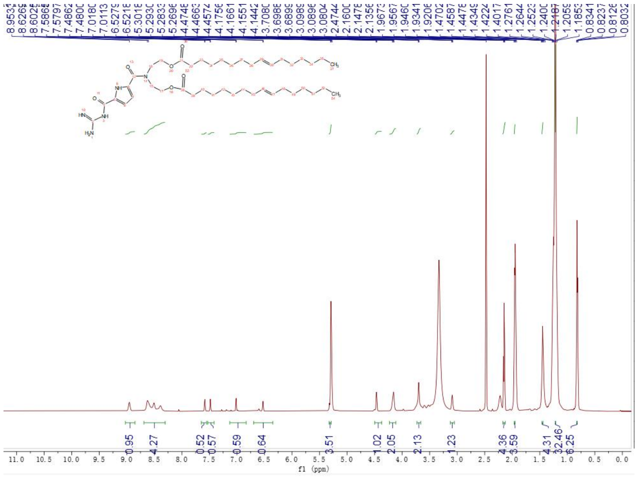

^{1}H NMR (600 MHz, DMSO-d_6) δ 8.95 (s, 1H), 8.69 – 8.30 (m, 4H), 7.58 (d, J = 4.0 Hz, 1H), 7.48 (d, J = 3.7 Hz, 1H), 7.01 (d, J = 4.0 Hz, 1H), 6.56 (dd, J = 45.6, 3.6 Hz, 1H), 5.30 (d, J = 5.3 Hz, 4H), 4.47 (t, J = 5.2 Hz, 1H), 4.16 (q, J = 6.5, 6.1 Hz, 2H), 3.70 (t, J = 5.4 Hz, 2H), 3.09 (t, J = 5.5 Hz, 1H), 2.15 (t, J = 7.3 Hz, 4H), 1.96 (s, 4H), 1.45 (d, J = 6.6 Hz, 4H), 1.22 (s, 32H), 0.82 (s, 6H).

MS (LC-MS): m/z calculated for $[C_{43}H_{73}N_5O_6]^+$: 756.5634; found $[M+H]^+$:756.5618

GL3:

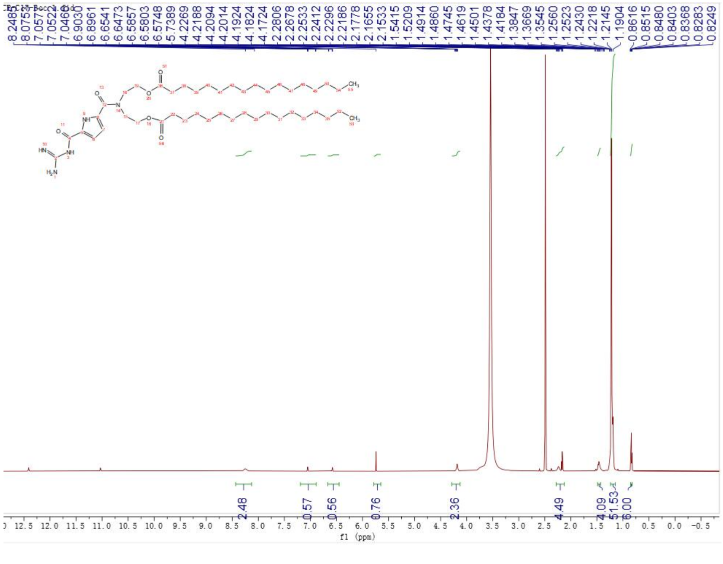

^{11}H NMR (600 MHz, DMSO-d_6) δ 8.25 (s, 2H), 7.19 – 6.89 (m, 1H), 6.67 – 6.45 (m, 1H), 5.74 (s, 1H), 4.19 (q, J = 5.7 Hz, 2H), 2.20 (dt, J = 45.8, 7.3 Hz, 4H), 1.46 (p, J = 7.2 Hz, 4H), 1.24 – 1.15 (m, 52H), 0.85 – 0.82 (m, 6H).

MS (LC-MS): m/z calculated for $[C_{45}H_{81}N_5O_6]^+$: 788.6259; found $[M+H]^+$:788.6254.

GL4:

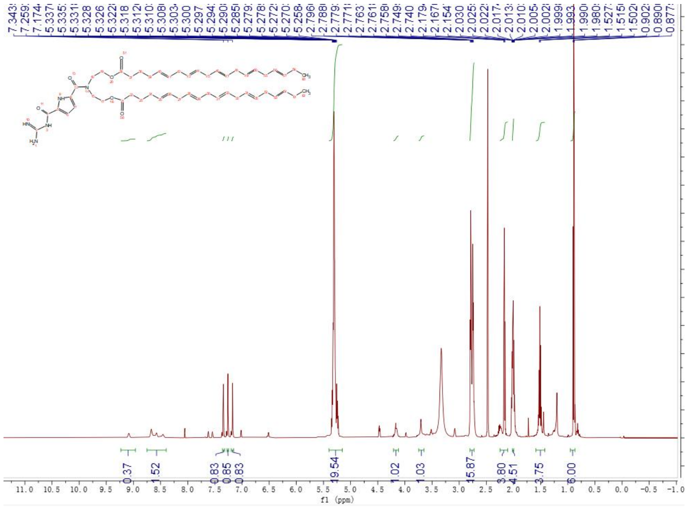

^{1}H NMR (600 MHz, DMSO-d_6) δ 8.75 – 8.40 (m, 2H), 7.34 (s, 1H), 7.26 (s, 1H), 7.17 (s, 1H), 5.40 – 5.15 (m, 20H), 4.21 – 4.12 (m, 1H), 3.74 – 3.65 (m, 1H), 2.77 (dt, J = 23.5, 5.4 Hz, 16H), 2.17 (t, J = 7.4 Hz, 4H), 2.02 – 2.00 (m, 5H), 1.52 (h, J = 7.2 Hz, 4H), 0.89 (t, J = 7.5 Hz, 6H).

MS (LC-MS): m/z calculated for [C51H73N5O6]+ : 852.5634; found [M+H]+ :852.5837

GL5:

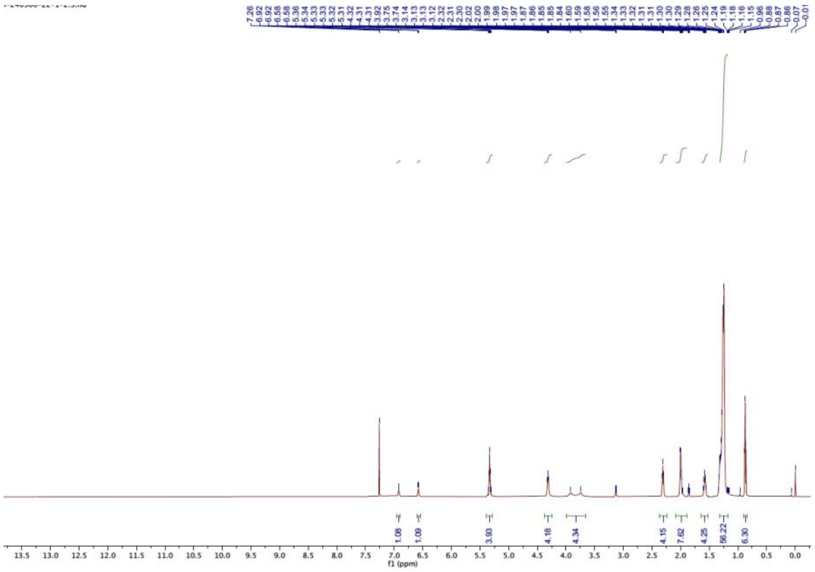

1H NMR (600 MHz, CDCl3) δ 6.92 (s, 1H), 6.58 (s, 1H), 5.36-5.31 (m, 4H), 4.32-4.31 (m, 4H), 3.92-3.74 (m, 4H), 2.32-2.30 (m, 4H), 2.02-1.97 (m, 8H), 1.60-1.55 (m, 4H), 1.34-1.15 (m, 56H), 0.88-0.86 (t, 6H).

MS (LC-MS): m/z calculated for [C55H97N5O6]+ : 924.7511; found [M+H]+ :924.7517.

GL6:

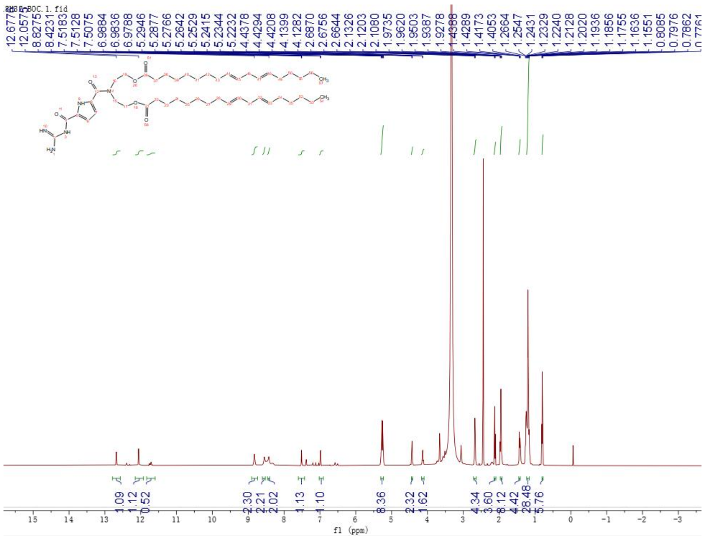

^{1}H NMR (600 MHz, DMSO-d_6) δ 9.01 (t, J = 6.1 Hz, 1H), 8.62 (d, J = 32.4 Hz, 2H), 8.48 (d, J = 63.3 Hz, 1H), 7.68 – 7.48 (m, 1H), 7.13 – 6.85 (m, 1H), 5.49 – 5.05 (m, 2H), 4.46 (t, J = 5.0 Hz, 1H), 3.81 – 3.63 (m, 1H), 3.54 (s, 1H), 3.18 – 2.96 (m, 1H), 2.23 (q, J = 15.0, 11.2 Hz, 1H), 2.14 (t, J = 7.3 Hz, 4H), 1.92 (ddt, J = 23.5, 11.9, 6.8 Hz, 2H), 1.68 (dp, J = 22.4, 6.6 Hz, 2H), 1.44 (q, J = 7.8, 7.1 Hz, 4H), 1.21 (d, J = 12.9 Hz, 40H), 0.82 (t, J = 6.8 Hz, 6H).

MS (LC-MS): m/z calculated for $[C47H77N5O6]^+$: 808.5946; found $[M+H]^+$:808.6439.

GL7:

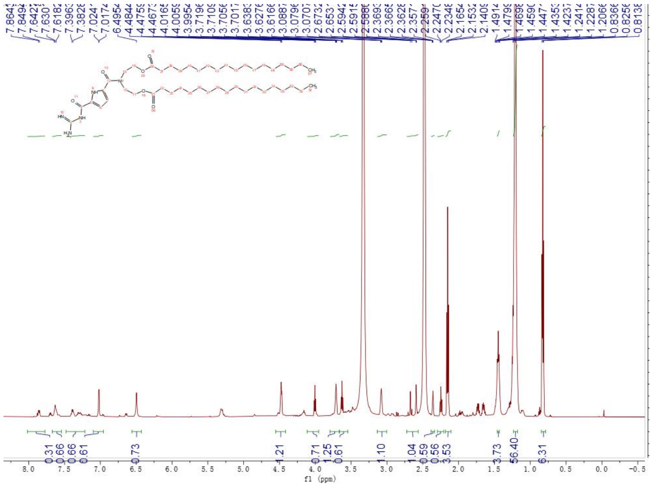

^{1}H NMR (600 MHz, DMSO-d_6) δ 7.62 (q, J = 7.8 Hz, 1H), 7.48 – 7.22 (m, 1H), 7.02 (t, J = 4.6 Hz, 1H), 6.50 (s, 1H), 4.55 – 4.41 (m, 1H), 4.01 (t, J = 6.3 Hz, 1H), 3.79 – 3.66 (m, 1H), 3.63 (t, J = 6.5 Hz, 1H), 3.08 (t, J = 5.4 Hz, 1H), 2.72 – 2.56 (m, 1H), 2.36 (d, J = 3.5 Hz, 1H), 2.25 (t,

J = 7.3 Hz, 1H), 2.15 (t, J = 7.4 Hz, 4H), 1.45 (t, J = 7.1 Hz, 4H), 1.21 (s, 56H), 0.83 (t, J = 6.8 Hz, 6H).

MS (LC-MS): m/z calculated for $[C47H85N5O6]^+$: 816.6573; found $[M+H]^+$:816.6591.

GL8:

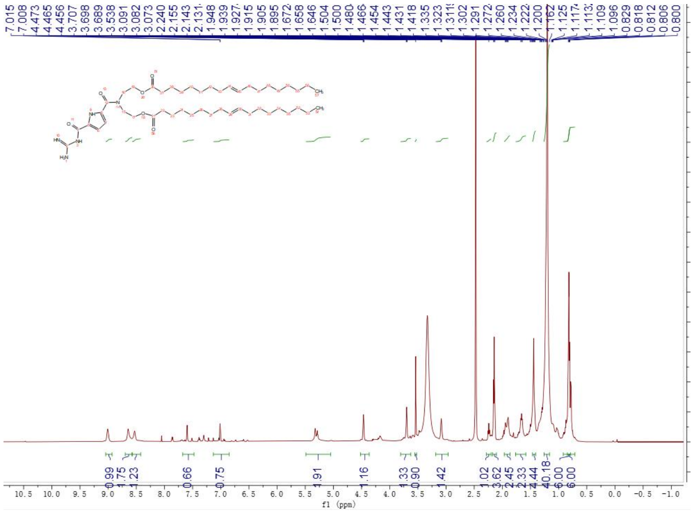

^{1}H NMR (600 MHz, DMSO-d_6) δ 7.68 – 7.48 (m, 1H), 7.13 – 6.85 (m, 1H), 5.49 – 5.05 (m, 2H), 4.46 (t, J = 5.0 Hz, 1H), 3.81 – 3.63 (m, 1H), 3.54 (s, 1H), 3.18 – 2.96 (m, 1H), 2.23 (q, J = 15.0, 11.2 Hz, 1H), 2.14 (t, J = 7.3 Hz, 4H), 1.92 (ddt, J = 23.5, 11.9, 6.8 Hz, 2H), 1.68 (dp, J = 22.4, 6.6 Hz, 2H), 1.44 (q, J = 7.8, 7.1 Hz, 4H), 1.21 (d, J = 12.9 Hz, 40H), 0.82 (t, J = 6.8 Hz, 6H).

MS (LC-MS): m/z calculated for $[C47H81N5O6]^+$: 812.6259; found $[M+H]^+$:812.6249.

GL9:

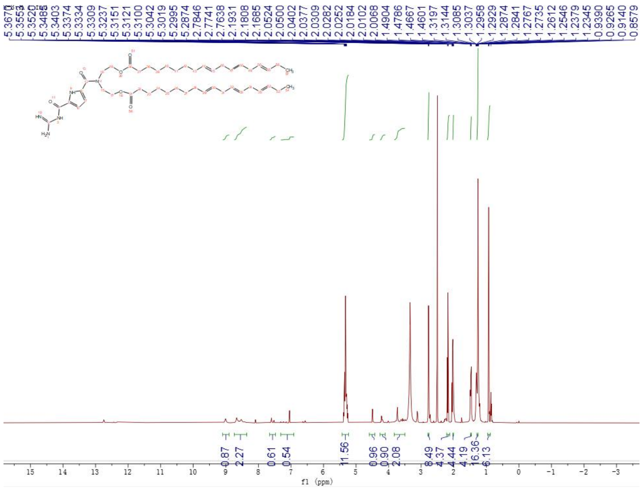

^{1}H NMR (600 MHz, DMSO-d_6) δ 9.01 (s, 1H), 8.74 – 8.36 (m, 2H), 7.65 – 7.48 (m, 1H), 7.31 – 6.91 (m, 1H), 5.43 – 5.23 (m, 12H), 4.60 – 4.42 (m, 1H), 4.26 – 4.10 (m, 1H), 3.83 – 3.50

(m, 2H), 2.77 (t, *J* = 6.2 Hz, 8H), 2.18 (t, *J* = 7.4 Hz, 4H), 2.03 – 2.01 (m, 4H), 1.47 (d, *J* = 7.1 Hz, 4H), 1.26 (d, *J* = 4.0 Hz, 16H), 0.93 (t, *J* = 7.5 Hz, 6H).

MS (LC-MS): m/z calculated for $[C47H73N5O6]^+$: 804.5633; found $[M+H]^+$:804.5773
